# Maternal contact and age-dependent succession influence the assembly of the calf rumen microbiome and virome

**DOI:** 10.1128/spectrum.01677-26

**Published:** 2026-07-22

**Authors:** Yoshiaki Sato, Yuma Uda, Yoshikazu Nagao

**Affiliations:** 1 Department of Agrobiology and Bioresources, School of Agriculture, Utsunomiya University 98317, Tochigi, Japan; 2 University Farm, School of Agriculture, Utsunomiya University 98317, Tochigi, Japan; Cleveland Clinic Lerner Research Institute, Cleveland, Ohio, USA

**Keywords:** rumen, virome, microbiome, cattle, metagenome

## Abstract

Early-life colonization of the rumen is particularly important; however, the processes by which microbial and viral communities are transmitted and developed remain poorly understood. Here, we present a genome-resolved investigation of the effects of maternal contact and age-dependent succession on the calf rumen microbiome and DNA virome by comparing calves raised with or without maternal contact across early life using the metagenome-assembled genomes (MAGs) and viral operational taxonomic units (vOTUs) reconstructed from whole- and virus-like particle metagenomes. Across longitudinal samples from calves and their mothers, we identified 694 MAGs and 30,479 vOTUs, substantially expanding current genome databases and revealing extensive microbial and viral novelty. Our analyses demonstrated that both prokaryotes and DNA viruses are shared between dams and calves, with greater sharing observed in calves raised with maternal contact than in calves raised without maternal contact. Notably, viral sharing between cow-calf pairs was markedly lower compared to prokaryotes, suggesting high turnover and rapid viral diversification. Age-associated analyses further revealed coordinated shifts in prokaryotes and their viruses, with dominant genera such as *Prevotella*, *Ruminococcus*, and *Fibrobacter,* and their corresponding viruses increasing after day 40. These findings indicate that the early-life rumen microbiome and DNA virome undergo substantial age-dependent succession and are associated with maternal contact, providing new insights into host-microbe-virus interactions during rumen development.

**IMPORTANCE:**

This study provides one of the first genome-resolved views of DNA viral community development during early rumen colonization in calves (from 1 week to 70 days of age) and reveals how maternal contact and age influence the establishment of the calf rumen microbiome and virome. By analyzing longitudinal samples from calves raised with or without their mothers, we show that prokaryotes and their viruses undergo coordinated, age-dependent succession. Our results demonstrate that maternal separation alters the assembly of the calf rumen microbiome, highlighting the influence of maternal contact during early-life rumen development. These findings underscore the high plasticity of the early-life rumen ecosystem and suggest that early management practices, such as maternal separation, can have lasting effects on rumen development. This work provides fundamental insights into the establishment and succession of the calf rumen microbiome and DNA virome during early life and may contribute to future microbiome manipulation studies.

## INTRODUCTION

The rumen microbiome plays an essential role in ruminant nutrition, productivity, and methane emissions by degrading complex plant polysaccharides and subsequently fermenting the resulting sugars into volatile fatty acids (VFAs) (1, 2). Early-life rumen colonization critically influences lifetime performance and host physiology, and several determinants of this process are increasingly understood (3). Among these factors, maternal contact is one of the key drivers of early-life rumen microbiota establishment (4, 5). However, few studies have examined microbial lineage transmission during early rumen development using genome-resolved metagenomics.

Within microbial species, substantial genomic and functional heterogeneity leads to differences in metabolism and ecological interactions (6, 7). Consequently, species-level overlap alone cannot distinguish vertical transmission from environmentally driven acquisition. Genome-resolved analyses of individual microbial lineages therefore provide clearer insights into microbial inheritance and acquisition. While human microbiome studies have demonstrated high-resolution maternal-infant microbial sharing (8), comparable genome-resolved analyses in the rumen microbiome remain scarce.

Beyond prokaryotes, the rumen virome (bacteriophages and archaeal viruses) is a critical yet understudied ecosystem component (9–11). Viruses can shape microbial communities, modulate prokaryotic population dynamics, and transfer auxiliary metabolic genes (9, 12) and may also influence host productivity traits, such as milk yield and meat production (11, 13). However, little is known about viral maternal transmission or its interaction with prokaryotic community establishment in ruminants. A comprehensive understanding of both prokaryotic and viral transmission is therefore needed to clarify host-microbe-virus interactions during early rumen development.

Another key question is the temporal dynamics of transmitted prokaryotes and viruses. Although previous studies have reported taxonomic and functional shifts in the rumen microbiome during calf development (14, 15), it remains unclear whether maternally transmitted prokaryotes and viruses are transient after birth or persist in establishing the rumen microbiome. Longitudinal sampling across early development can distinguish transient colonization from stable inheritance and assess shifts between maternal transmission and environmental acquisition.

In this study, we investigated the effects of maternal contact and age-dependent succession on rumen prokaryotic and viral communities during the pre-weaning period using genome-resolved metagenomics of whole-community and virus-like particle (VLP) fractions. Using longitudinal samples from calves raised either with or without maternal contact, we aimed to (i) characterize rumen prokaryotic and viral diversity, (ii) assess metagenome-assembled genomes (MAGs) and viral operational taxonomic units (vOTUs) shared between dams and calves, and (iii) investigate age-dependent changes in rumen prokaryotic and viral communities. Together, these analyses provide a genome-resolved view of rumen prokaryotic and DNA viral communities during early life and highlight the associations between maternal contact and rumen microbiome development in calves.

## RESULTS

### Expanding diversity of the rumen prokaryotic and viral genomes in the cow and early-life calf

We investigated the rumen microbiome of Japanese Black calves at TP1 (6–8 days), TP2 (38–40 days), and TP3 (68–70 days) and of their mothers sampled at TP1 (M) by whole- and VLP-metagenomic sequencing (Table S1). Calves were either raised with their mothers (C, *n* = 5) or separated immediately after birth (S, *n* = 6). In total, 694 MAGs classified as medium- (≥50% completeness and ≤10% contamination) or high-quality (≥90% completeness and ≤5% contamination) using CheckM2 (16) were reconstructed after dereplication with dRep (17) at average nucleotide identity (ANI) ≥99%. All MAGs were assigned at the phylum level, spanning 15 bacterial and 2 archaeal phyla, while 3 and 252 MAGs were not assigned at the genus and species level by GTDB-Tk (18), respectively, indicating putative novel taxa (Table S2). Bacillota (*n* = 295) and Bacteroidota (*n* = 254) were the dominant phyla (Fig. S1; Table S2). At the genus level, *Prevotella* was most frequent (69 MAGs), followed by *Cryptobacteroides* (39 MAGs), *Succiniclasticum* (25 MAGs), and *Physcousia* (23 MAGs) (Table S2). Carbohydrate-active enzymes (CAZymes) annotation revealed that dominant genera, particularly *Prevotella*, *Cryptobacteroides*, and *Physcousia*, were enriched in glycoside hydrolases (GHs) involved in plant polysaccharide degradation (e.g., GH5, GH9, and GH43; Fig. S2A and B), highlighting their contribution to rumen lignocellulose degradation.

**Fig 1:**
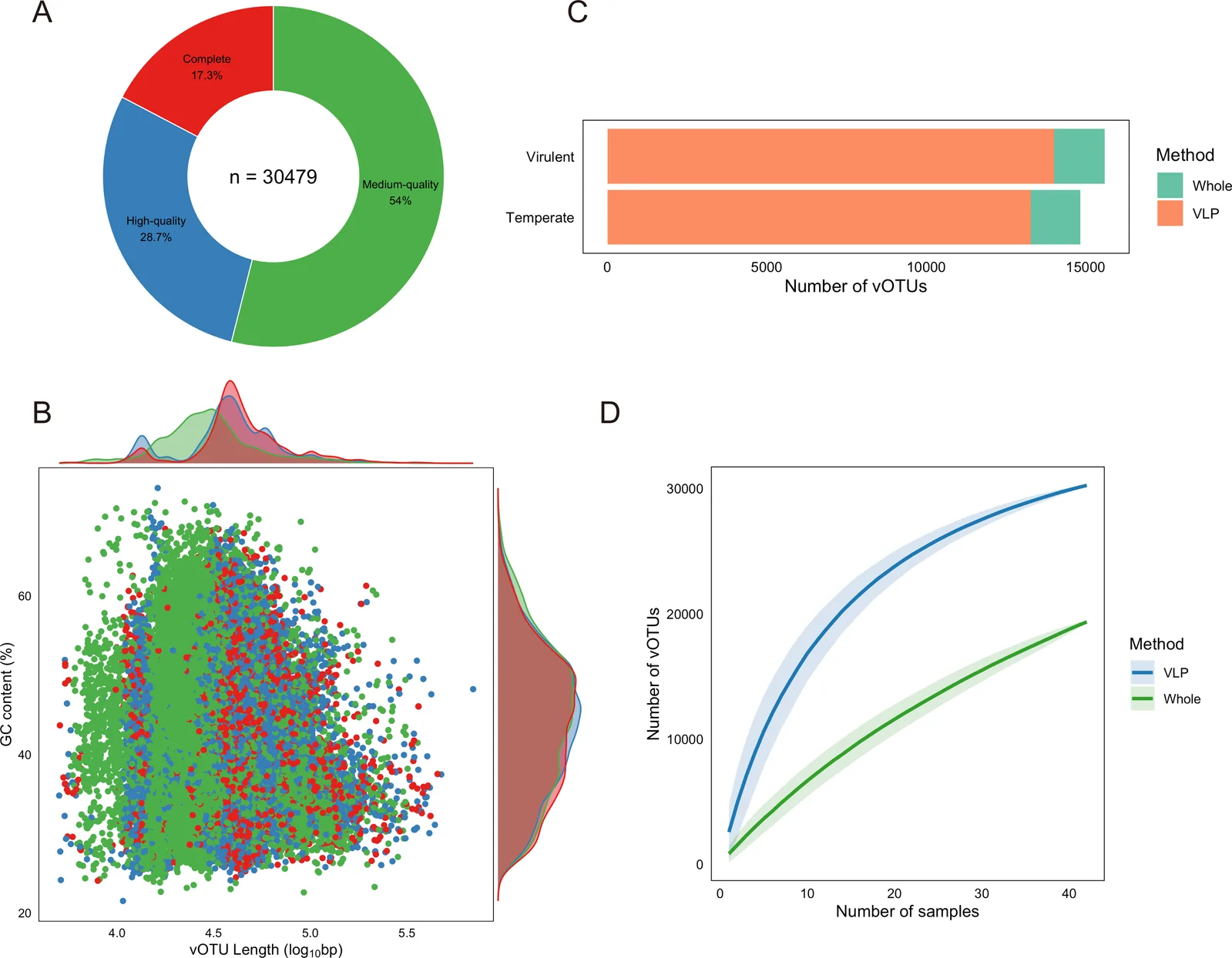
Overview of the reconstructed vOTUs in RCCV. (**A**) Donut chart showing the proportion of vOTU quality categories assessed using CheckV. (**B**) Scatter plot of genome length versus GC content. Red, blue, and green dots represent complete, high-quality, and medium-quality vOTUs, respectively. (**C**) Proportion of temperate and virulent viruses. (**D**) Accumulation curves of vOTUs.

VLP-metagenomic sequencing generated an average of 54.2 ± 13.8 million reads per sample after filtering. Viral contigs (>5 kb) were identified from both VLP- and whole-metagenomic assemblies using geNomad (19), yielding 273,140 contigs. After quality assessment with CheckV (20) and clustering at 95% identity and 85% coverage, 30,479 vOTUs were retained, including 5,277 complete (17.3%), 8,758 high-quality (≥90% completeness; 28.7%), and 16,444 medium-quality (50%–90% completeness; 54.0%) genomes, hereafter referred to as the rumen cow-calf viral genomes (RCCV) (Fig. 1A; Table S3). Among them, 89.7% (*n* = 27,327) and 10.3% (*n* = 3,152) of vOTUs were obtained from VLP- and whole-metagenomic data, respectively. Their genome lengths averaged 41.8 ± 33.9 kb, ranging from 5,006 to 695,497 bp, and GC contents averaged 44.2% ± 9.1%, ranging from 21.5% to 73.62% (Fig. 1B; Table S3). Notably, 30,395 vOTUs (99.7%) were assigned to the class *Caudoviricetes*, and 14,830 vOTUs (48.7%) were predicted to be temperate viruses (Fig. 1C). An accumulation curve revealed that the number of vOTUs did not approach saturation, indicating that RCCV did not comprehensively capture the rumen DNA virome diversity in the data set (Fig. 1D).

Gene-sharing network analysis using vConTACT2 (21) clustered RCCV into 11,365 viral clusters (VCs), including 8,987 singletons, approximating genus-level groupings (Fig. S3). Only 70 VCs were clustered with RefSeq viral genomes, suggesting that most VCs were putative novel genera (Table S3). Additionally, we compared the vOTUs to references rumen viral genomes, including RVD (*n* = 41,738) (9), RVG (*n* = 8,232) (10), and JBRV (*n* = 22,942) (11), which contain complete, high-quality, and medium-quality viral genomes reconstructed from rumen metagenomic data sets, using clustering at 95% nucleotide identity and 85% alignment coverage. As a result, only 36 (0.12%), 45 (0.15%), and 96 vOTUs (0.31%) were clustered into vOTUs in RVD, RVG, and JBRV, respectively, suggesting that RCCV includes many novel rumen viral genomes (Table S3).

Host prediction based on CRISPR spacers extracted from prokaryotic genomes of ruminant gastrointestinal tracts (22) and the reconstructed MAGs identified prokaryotic hosts of 4,267 vOTUs (14.0%), primarily within Bacteroidota (*n* = 1,724) and Bacillota (*n* = 1,604) (Fig. S4A). Notably, 57 vOTUs were associated with archaeal hosts, with most of them associated with Methanobacteriota, a dominant methanogen in the rumen. Among the prokaryotic host-predicted vOTUs, 815 vOTUs (19.1%) were generalists, meaning they were linked to multiple genera, while the others were specialists, associated with a single genus. Among these specialists, the most common genus was *Prevotella* (*n* = 416), followed by *Cryptobacteroides* (*n* = 213), *Treponema_D* (*n* = 152), *Sodaliphilus* (*n* = 121), and *Fibrobacter* (*n* = 110) (Fig. S4B), all representing the common genus in the rumen early in life and in adults (7, 23, 24). Among the viruses associated with archaea, the hosts belonged to *Methanobrevibacter, Methanocatella,* and *Methanosphaera* (Table S3).

### Difference in the rumen DNA virome between whole and VLP metagenomes

**Fig 2:**
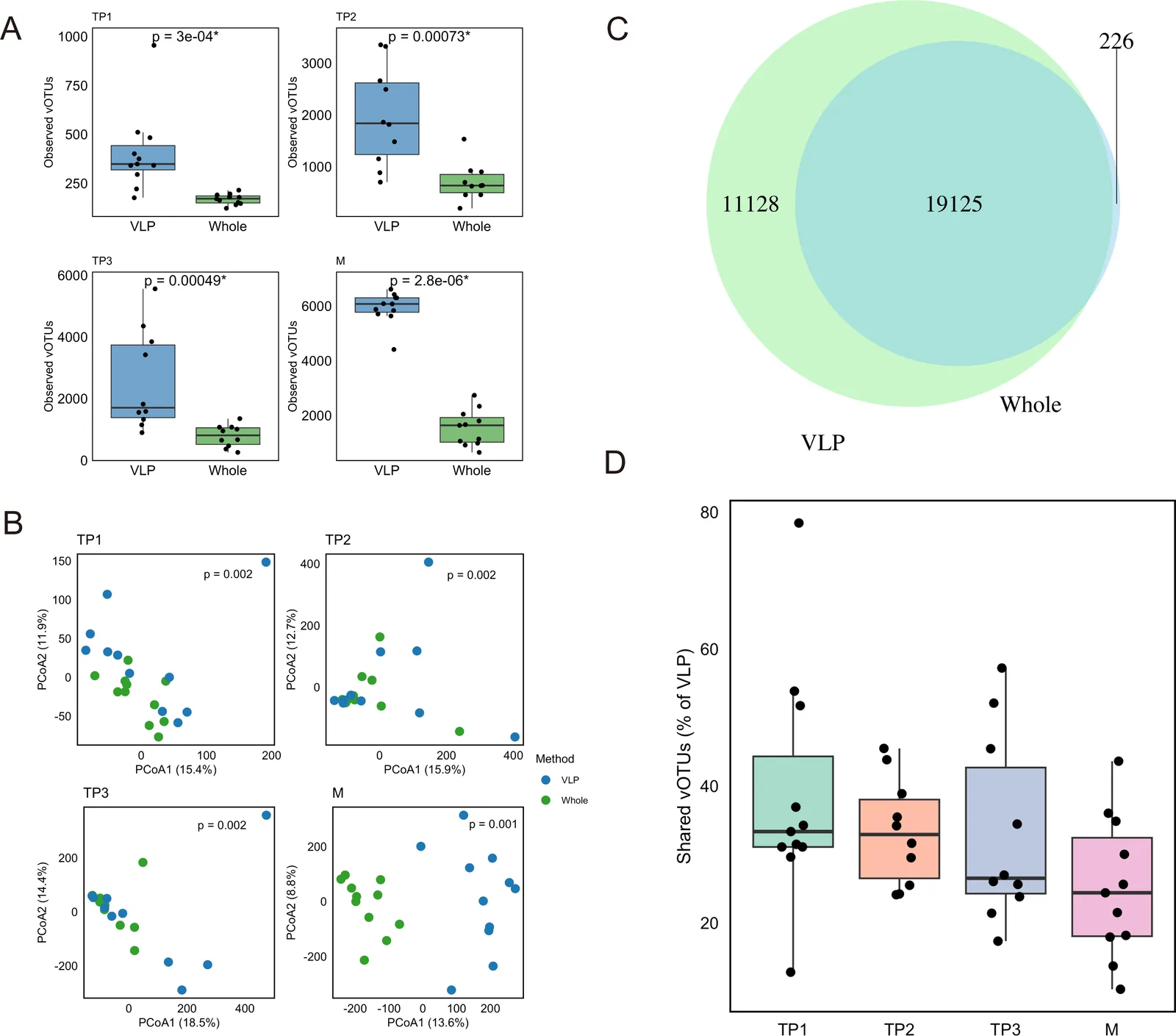
Difference of the rumen virome between whole and VLP-metagenome sequencing. (**A**) Alpha-diversity and (**B**) beta-diversity at each time point (TP1–TP3) and in the mother. (**C**) Venn diagram showing the overlap between whole-metagenome and VLP-metagenome sequencing. (**D**) Percentage of shared vOTUs between whole-metagenome and VLP-metagenome sequencing.

Previous studies have reported differences in virome diversity between whole and VLP metagenomes in the human gut and rumen (9, 25). Here, we compared the rumen DNA virome diversity between the two methods. The number of vOTUs was significantly higher in VLP metagenomes than in whole metagenomes across mothers and all calf time points (*P* < 0.05, Fig. 2A), and PERMANOVA confirmed a significant effect of sequencing method at all time points (*P* < 0.05; Fig. 2B). Additionally, 11,128 and 226 vOTUs were detected exclusively in VLP and whole metagenomes, respectively (Fig. 2C), and only 32.4% ± 1.44% of vOTUs were shared within individual samples (Fig. 2D). These results indicate that whole- metagenome captures only a limited subset of rumen viral populations, consistent with previous observations in soil (26) and rumen virome (9). Together, our findings highlight the advantage of VLP-metagenome sequencing for capturing rumen viral diversity and suggest that whole-metagenome substantially underestimates viral richness.

### Temporal development of the early-life rumen microbiome

**Fig 3:**
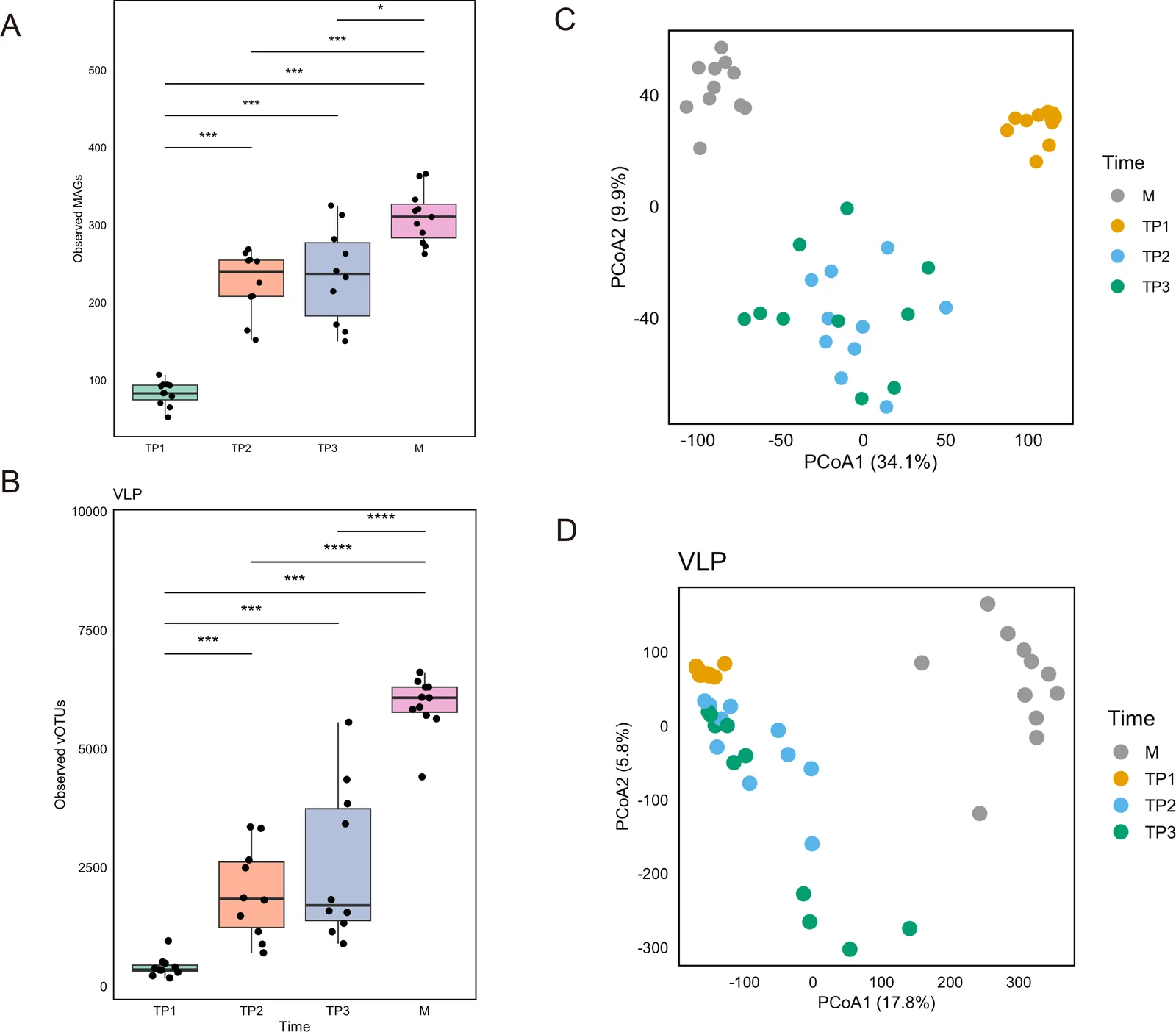
Time-dependent changes in the rumen prokaryotic and viral communities. (**A**) The number of observed MAGs and (**B**) the number of observed vOTUs based on VLP metagenomes. Statistical significance was assessed by Wilcoxon rank-sum tests with FDR correction and indicated as follows: FDR ≤ 0.05 (*), FDR ≤ 0.01 (**), FDR ≤ 0.001 (***), and FDR ≤ 0.0001 (****). PCoA plots of (**C**) prokaryotic community structures based on MAGs and (**D**) DNA viral community structures based on vOTUs from VLP metagenomes. Euclidean distances of CLR-transformed TPM values (TPM + 0.01) were used.

To examine the rumen microbiome dynamics, whole-metagenomic reads were mapped to MAGs for prokaryotes, and reads from whole and VLP metagenomes were mapped to RCCV vOTUs for viruses. The number of observed MAGs in TP2 and TP3 was significantly higher than that in TP1, but still lower than that in M (false discovery rate [FDR] < 0.05, Fig. 3A). Similar trends were observed in the number of observed vOTUs in both whole and VLP metagenomes (FDR < 0.05; Fig. 3B; Fig. S5A). Community composition differed significantly among all time points and between calves and mothers for both prokaryotes and viruses (pairwise PERMANOVA, FDR < 0.05; Fig. 3C and D; Fig. S5B and C). The most pronounced shift occurred between TP1 and TP2, as indicated by significantly lower proportions of shared MAGs and vOTUs compared with TP2–TP3 within the same animals (*P* < 0.01; Fig. S5D and E). Together, these results show that rumen prokaryotic and viral communities undergo rapid early-life restructuring, and although richness increased over time, communities remained distinct from adults, indicating incomplete maturation by 70 days of age.

**Fig 4:**
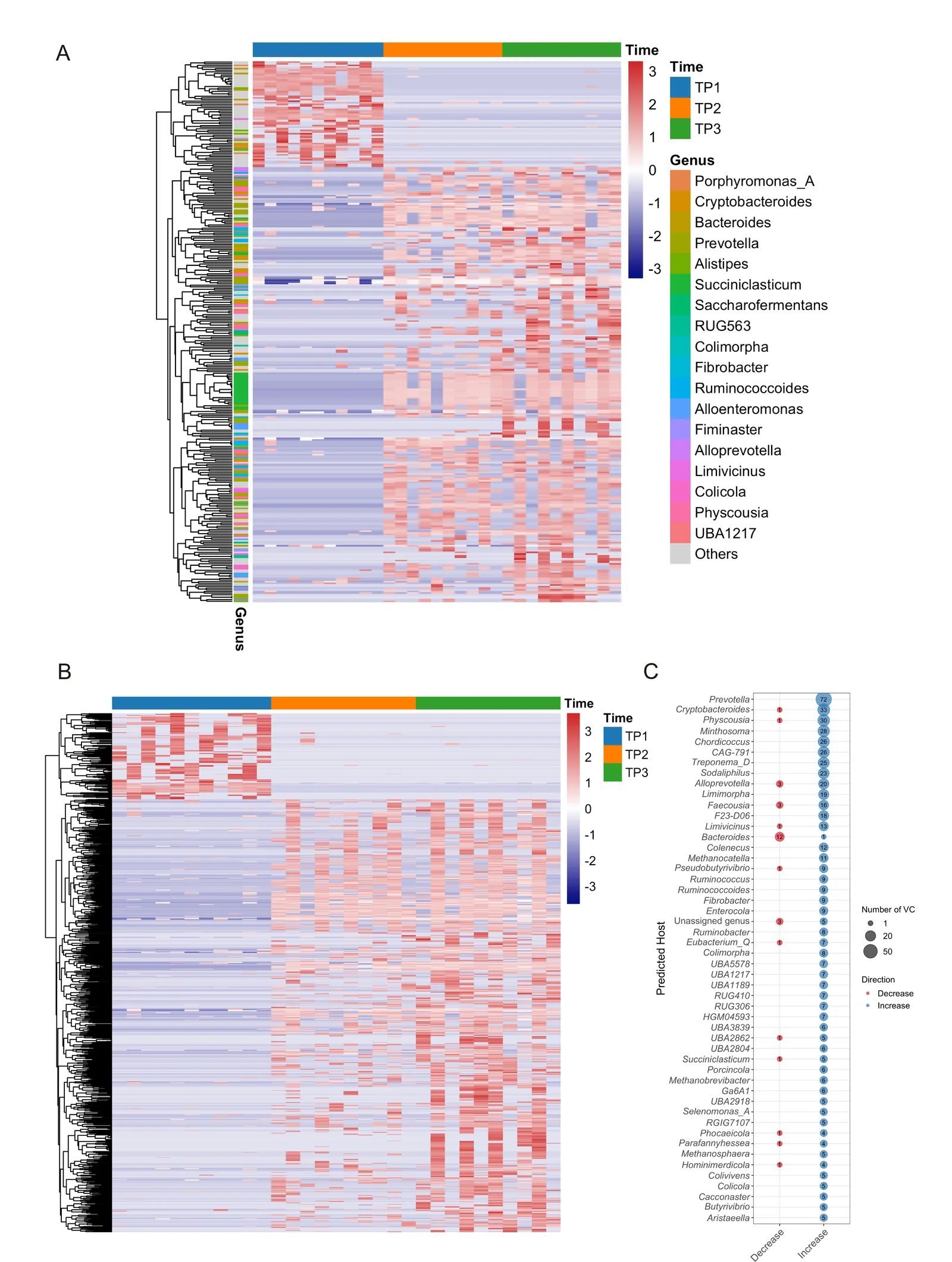
Age-dependent changes in rumen MAGs and VCs during the early life of calves. Heatmaps of significantly changed MAGs (**A**) and VCs identified from VLP metagenomes (**B**) in an age-dependent manner. Statistical analysis was performed using MaAsLin2 (FDR < 0.05). Values represent CLR-transformed TPM (TPM + 0.01). (**C**) Predicted prokaryotic hosts of VCs are significantly influenced by age-dependent effects. The top 50 host genera were selected.

To identify age-associated changes in the rumen microbiome, we applied MaAsLin2 (27). In total, 267 MAGs showed significant increases with age, including *Prevotella* (*n* = 51), *Cryptobacteroides* (*n* = 25), *Succiniclasticum* (*n* = 25), *Physcousia* (*n* = 14), *Colicola* (*n* = 14), *Alloenteromonas* (*n* = 13), *Fibrobacter* (*n* = 11), and *Ruminococcus* (*n* = 3) (Fig. 4A). Additionally, one archaeal MAG belonging to the genus JAKSHX01 also increased with age. However, because only three archaeal MAGs were reconstructed, archaeal diversity in the calf rumen may not have been comprehensively captured, and archaeal taxa with age-dependent dynamics may have been overlooked. In contrast, 65 MAGs were significantly decreased, such as *Alistipes* (*n* = 5), *Bacteroides* (*n* = 5), and *Porphyromonas_A* (*n* = 4) (FDR < 0.05) (Fig. 4A). Most of these changes occurred during the transition from TP1 to TP2, consistent with the overall community shifts.

For viruses, 993 VCs showed significant age-associated changes (VLP, *n* = 766; whole, *n* = 51; both, *n* = 176), with 805 increasing and 188 decreasing over development (Fig. 4B; Fig. S6). After prokaryotic host prediction, we observed parallel age-dependent dynamics between viruses and their hosts. For instance, VCs associated with *Prevotella* (*n* = 72), *Cryptobacteroides* (*n* = 33), *Physcousia* (*n* = 30), *Fibrobacter* (*n* = 9), and *Ruminococcus* (*n* = 9) increased with age, whereas those associated with *Bacteroides* (*n* = 12) decreased (Fig. 4C). These results suggest coordinated shifts in both viruses and their hosts during rumen development. For archaeal viruses, VCs linked to *Methanocatella* (*n* = 11), *Methanobrevibacter* (*n* = 6), and *Methanosphaera* (*n* = 5) increased in an age-dependent manner.

To validate this observation, we investigated the correlations between viruses and their predicted hosts. Twelve genera were selected based on consistent age-related trends in both viruses and host MAGs. Significant correlations (*P* < 0.05) were observed in several genera, including *Prevotella*, *Cryptobacteroides*, *Saccharofermentans*, *Bacteroides*, and *Alloprevotella*, reinforcing the hypothesis that rumen viruses and their hosts are co-established in an age-dependent manner (Fig. S7). However, not all virus-prokaryote pairs showed significant correlations. It should be noted that only 14% of the vOTUs could be assigned to prokaryotic hosts, and only 694 MAGs were reconstructed, indicating potential underestimation of both viral and prokaryotic diversity. In fact, MAGs and vOTUs derived from well-reconstructed genera, such as *Prevotella* and *Cryptobacteroides*, showed stronger correlations, suggesting that increased recovery of MAGs and viral genomes is critical for more accurate validation in future studies.

### Mother-calf separation effects on the rumen microbiome in early life

**Fig 5:**
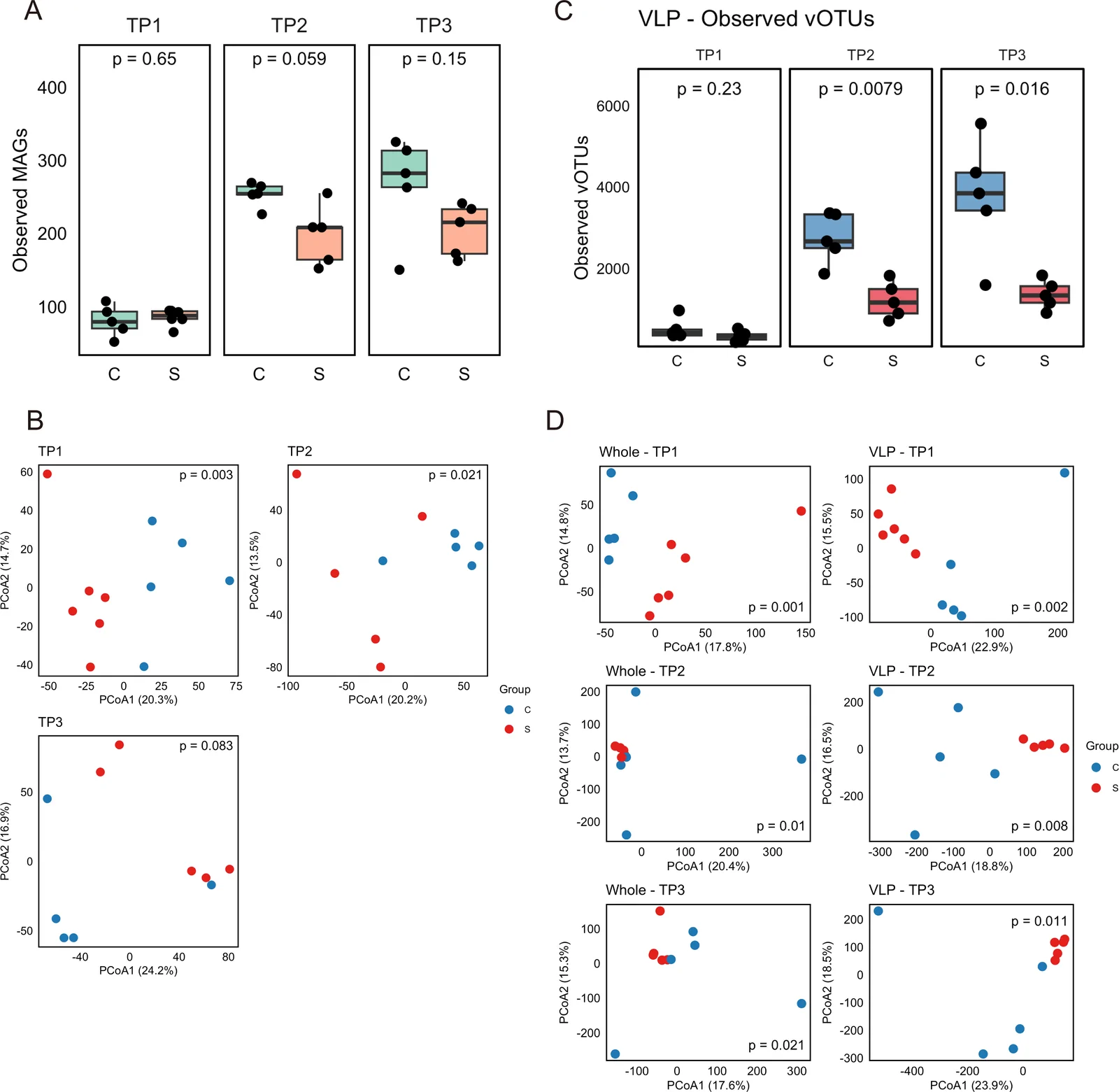
Effects of cow-calf separation on prokaryotic and viral diversity. (**A**) The number of observed MAGs and (**C**) the number of observed vOTUs identified from VLP metagenomes. PCoA plots of (**B**) prokaryotic and (**D**) DNA viral community structures based on whole and VLP metagenomes. Euclidean distances of CLR-transformed TPM values (TPM + 0.01) were used.

For the prokaryotic community, the number of observed MAGs was 22.0% lower in group S at TP2 (C vs S: 253.2 vs 197.4; *P* = 0.059), with a 23.3% reduction also observed at TP3 (266.6 vs 204.6; *P* = 0.15) despite lacking statistical significance (Fig. 5A). No significant difference was shown at TP1. PCoA showed distinct clustering between groups at TP1 and TP2 (*P* < 0.05), but not at TP3 (Fig. 5B). Similarly, for the virome, the number of vOTUs was significantly lower in group S at TP2 (whole and VLP) and TP3 (VLP only) (*P* < 0.05) (Fig. 5C; Fig. S8), and PCoA revealed clear group separation at all time points (*P* < 0.05) (Fig. 5D).

To investigate group-specific colonization patterns of prokaryotic and viral communities, we identified differentially abundant MAGs and VCs between groups using DESeq2 (28). In total, 67 MAGs and 1,008 VCs were significantly enriched in either group at any time point (FDR < 0.05; |log2 fold change| > 2). Notably, at TP1, a larger number of MAGs and VCs were more abundant in group S than in group C, whereas this trend was reversed at TP2 and TP3 (Fig. S9A and B).

**Fig 6:**
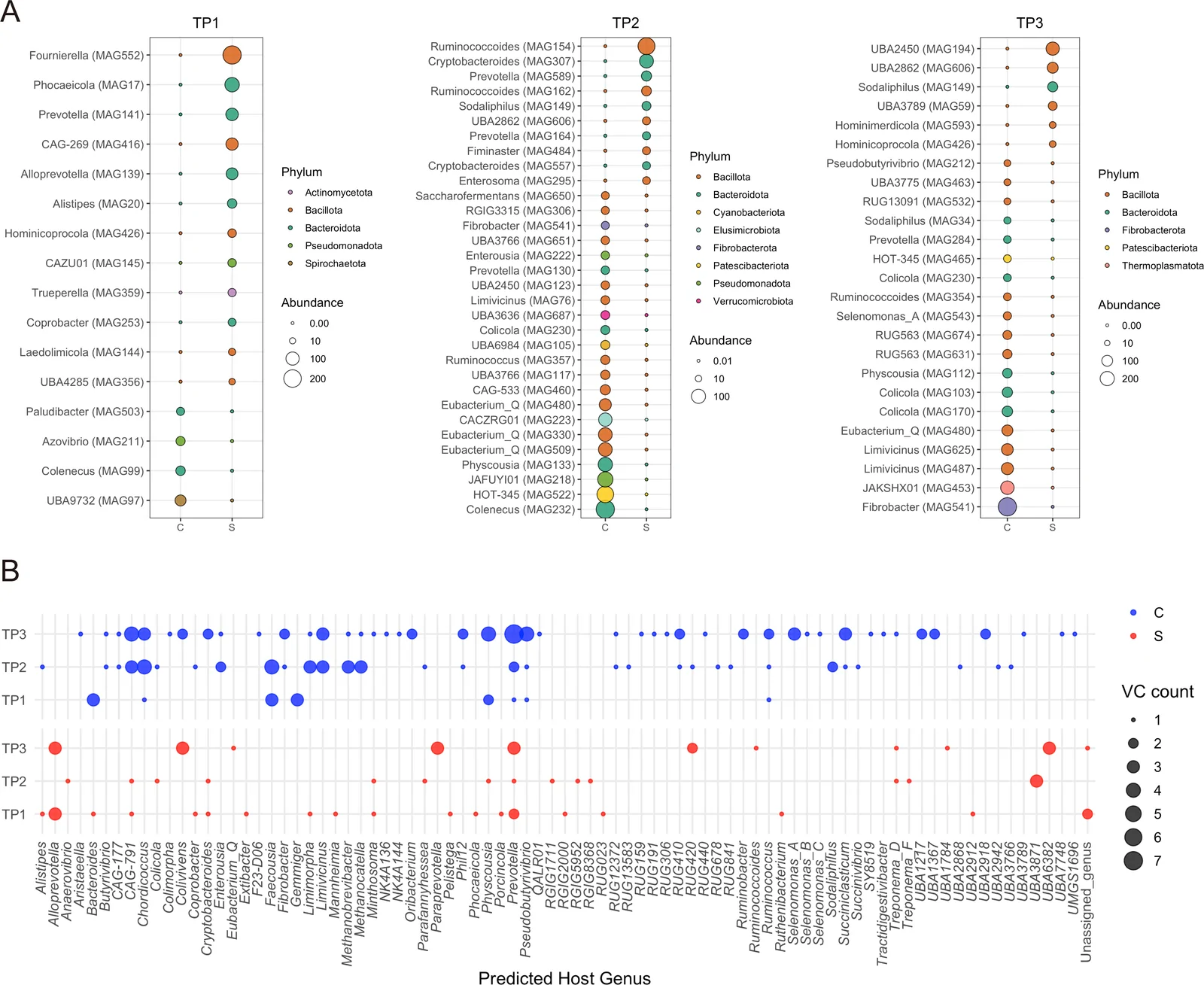
Differential taxa between cow-calf separation and non-separation groups. (**A**) Genera of significantly different MAGs between groups C and S at TP1, TP2, and TP3. (**B**) Predicted prokaryotic hosts of significantly different VCs between groups C and S at TP1, TP2, and TP3. Statistical tests were performed using DESeq2 (FDR < 0.05 and |log2 fold change| > 2).

At TP1, group S had more MAGs from the phyla Bacteroidota and Bacillota, including genera belonging to *Fournierella*, *Phocaeicola*, and *Prevotella*, whereas group C had only a few enriched MAGs such as *Azovibrio* and *UBA9732* (Fig. 6A). By TP2 and TP3, the trend reversed, with fibrolytic genera *Ruminococcus* and *Fibrobacter* becoming significantly more abundant in group C (Fig. 6A). These genera are essential for cellulose and hemicellulose degradation in the rumen (29), suggesting that maternal contact may promote colonization by functionally important microbes during early life. Correspondingly, alpha-diversity was increased by over 20% at TP2 and TP3 by maternal contact. Moreover, group C exhibited greater phylogenetic diversity across MAGs, encompassing members of Fibrobacterota, Spirochaetota, Verrucomicrobiota, and Actinomycetota, while group S remained dominated by Bacteroidota and Bacillota. These findings suggest that maternal contact may facilitate the establishment of a more phylogenetically diverse and functionally rich rumen microbiota during early development. Although digestion was not directly measured, the greater body weight gain observed in group C during the experiment (TP1–TP3) (*P <* 0.05) supports a potential link between microbial colonization and host growth (Fig. S10).

Consistent with this, VCs enriched in group C were predicted to infect a broader range of prokaryotic hosts (Fig. 6B). Viruses targeting *Ruminococcus*, *Fibrobacter*, *Selenomonas_A*, and *Succiniclasticum* were significantly more abundant in group C at TP2 and TP3, as were those targeting *Methanobrevibacter* and *Methanosphaera*. Interestingly, VCs infecting *Prevotella* were enriched in both groups at different time points, suggesting that maternal contact is not the sole factor shaping all virus-prokaryote associations.

**Fig 7:**
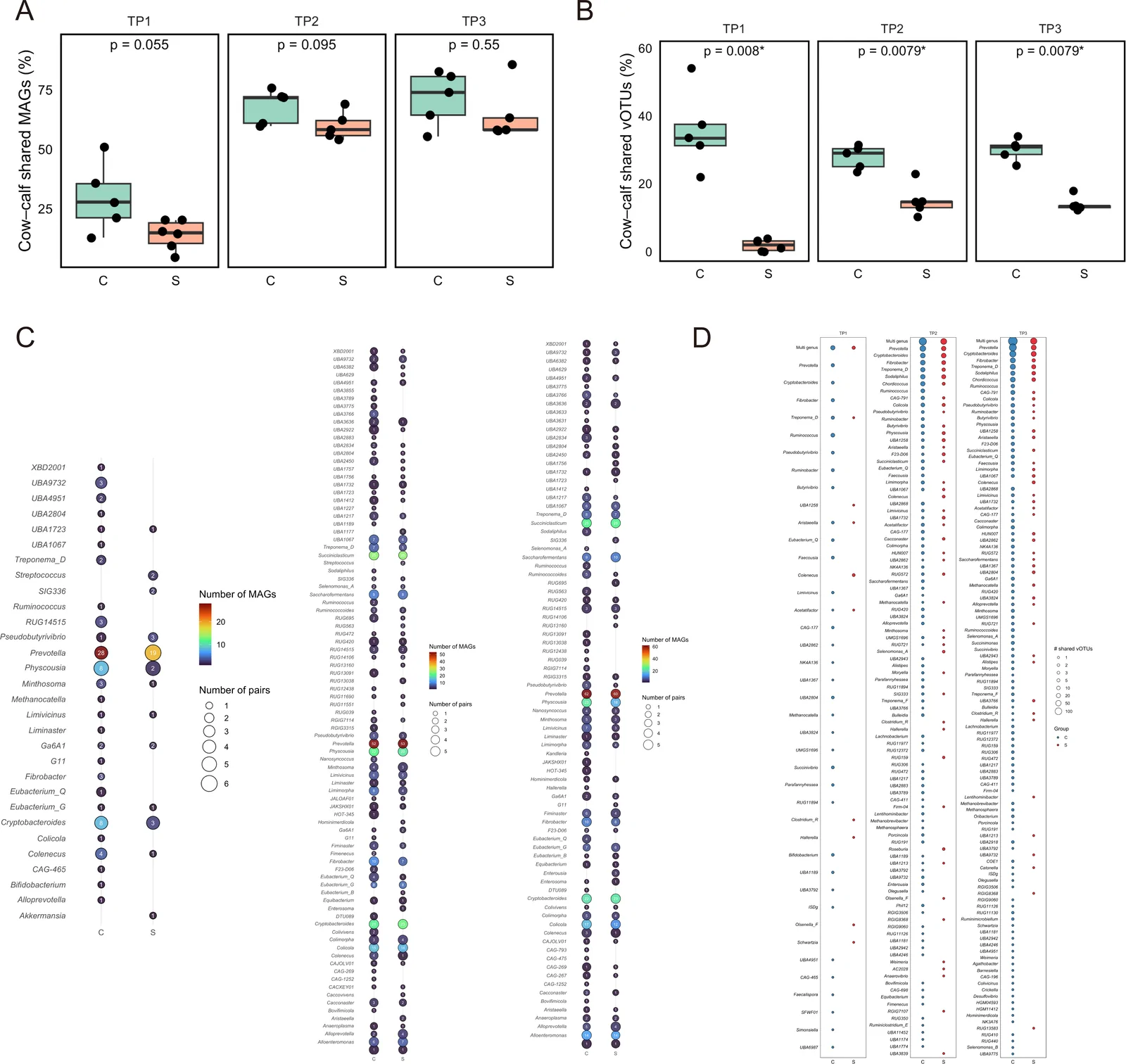
Shared microbiome between cow-calf pairs in separation and non-separation groups. Shared percentages of (**A**) MAGs and (**B**) vOTUs based on VLP metagenomes between each cow-calf pair in groups C and S. (**C**) Shared MAGs at the genus level. (**D**) Predicted prokaryotic hosts of shared vOTUs based on VLP metagenomes.

To investigate maternal transmission, we assessed MAG and vOTU sharing between dams and calves. For MAGs, group S tended to have lower sharing percentages at TP1 and TP2 (*P* = 0.055 and 0.095), with no difference at TP3 (Fig. 7A). However, vOTU sharing based on VLP metagenomes was consistently higher in group C at all time points (*P* < 0.01), most notably at TP1 (35.5% in C vs 1.78% in S) (Fig. 7B), indicating that viral transmission primarily occurs through postnatal contact. Moreover, this maternal influence on the rumen virus appears to persist until approximately 70 days of age.

Next, we analyzed which MAGs were shared between each cow-calf pair. At TP1, MAGs shared exclusively in group C, group S, and both groups numbered 54, 11, and 28, respectively (Fig. S11A). While the total number of shared MAGs increased over time, group C consistently exhibited a higher number of exclusively shared MAGs: at TP2, C = 78, S = 38, and both = 227; and at TP3, C = 96, S = 27, and both = 240 (Fig. S11B and C). These findings suggest that maternal contact facilitates broader and potentially earlier microbial transmission from dams to calves.

Taxonomic profiling of shared MAGs revealed that group C calves acquired a more diverse range of bacterial genera from their dams, especially at TP1 (Fig. 7C). These included functionally important fibrolytic bacteria such as *Ruminococcus* and *Fibrobacter*. In contrast, shared MAGs in group S were largely limited to common core taxa such as *Prevotella* and *Cryptobacteroides*. Notably, *Ruminococcus* was consistently shared only in group C across all time points, while *Fibrobacter* was initially exclusive to group C at TP1 but also detected in group S by TP2 and TP3. Although the overall trend persisted across time points, these differences became less pronounced at TP2 and TP3, suggesting that environmental exposure may gradually compensate for the lack of maternal contact to some extent.

Finally, predicted prokaryotic hosts of shared vOTUs were more taxonomically diverse in group C at all time points, more clearly so than for MAGs (Fig. 7D; Fig. S12). *Ruminococcus* vOTUs and those targeting dominant methanogens such as *Methanobrevibacter* and *Methanosphaera* were shared only in group C. These results highlight the stronger and more persistent role of maternal contact in shaping the early-life DNA virome compared to prokaryotic microbiota. Such influence may also regulate prokaryotic community dynamics, contributing to lignocellulose degradation and methane production in the developing rumen.

## DISCUSSION

Early-life rumen microbiome plays a critical role in establishing efficient fermentation, supporting immune system development, and influencing host energy and nutrient metabolism. However, our current understanding has largely focused on general patterns of microbial composition, with limited knowledge about how rumen microbiota are established and how they change during early calf development. In this study, we present a genome-resolved investigation of the effects of maternal contact and age-dependent succession on the calf rumen microbiome and DNA virome, encompassing both MAG- and vOTU-level analyses. Our results reveal that the DNA virome undergoes parallel succession with the prokaryotic community and that both prokaryotic and viral communities are influenced by maternal separation. Additionally, by reconstructing 694 MAGs and 30,479 vOTUs, we provide a genomic resource for studying the assembly and succession of the rumen microbiome.

Our MAGs and vOTUs substantially expand currently available genome databases, as most MAGs could not be assigned to GTDB at the species level, and most vOTUs did not cluster with sequences in RefSeq or in recently established rumen viral data sets such as RVD (9), RVG (10), and JBRV (11). This expansion likely reflects the inclusion of both early-life calves and adult cows in our study, whereas most previous rumen virome studies have primarily focused on adult cattle (7, 9–11, 30). Given the pronounced differences in rumen microbiome composition between calves and adults (4, 31) and further demonstrated in this study, our database provides an essential reference for future investigations of the rumen microbiome across developmental stages. Moreover, for RCCV, we identified putative prokaryotic hosts for 4,267 vOTUs (14.0%), providing valuable insights into rumen virus-prokaryote interactions. As the majority of prokaryotic hosts remain unresolved, expanding host-linkage predictions represents an important future challenge. Taken together, this integrated catalog establishes a foundational resource for investigating the early-life rumen microbiome. Importantly, unlike RVD, our use of VLP sequencing enabled greater sensitivity in recovering viral diversity, potentially capturing rare members that are often missed by whole-metagenome approaches.

Additionally, this study investigated the age-dependent change in both prokaryotic and viral communities. Microbial communities at TP1 were markedly different from those at TP2 and TP3, potentially reflecting the transition that occurs as calves begin to actively consume solid feed and their rumen microbiota gradually shift toward an adult-like composition (32). For example, major bacterial taxa such as *Prevotella* and *Cryptobacteroides*, which have a high number of GHs (Fig. S2A), increased substantially from TP2 onward. Strikingly, viruses infecting these bacteria exhibited similar temporal patterns, indicating that the development of the DNA virome parallels that of their prokaryotic hosts. To our knowledge, this is the first evidence demonstrating that the rumen DNA virome undergoes coordinated succession alongside the prokaryotic community during early life. Given that viruses infect and regulate their prokaryotic hosts, these results suggest that the rumen DNA virome may actively contribute to the functional maturation of the rumen, including digestion and degradation processes, by modulating host metabolism and abundance.

Another key finding of this study was the profound effect of maternal separation on both prokaryotic and viral communities. While cow-calf contact has previously been shown to influence the rumen microbiome (5), our results extend these observations by demonstrating the transmission of rumen microorganisms at the levels of MAGs and vOTUs. Our results demonstrated that calves separated from their dams tended to exhibit lower MAG richness and a reduced proportion of shared MAGs with their dams (*P* < 0.1). In contrast, the effect was statistically significant for viruses: the proportion of shared vOTUs between cow-calf pairs was drastically reduced in the separation group, and at TP1, it was nearly absent (*P* < 0.05). This result suggests that transmission of prokaryotes and viruses is not substantially achieved during delivery itself and that postnatal maternal-associated exposures likely play a major role in transmission. Importantly, our findings highlight that viruses, in addition to prokaryotes, are strongly influenced by maternal contact, underscoring that the early-life rumen DNA virome is highly dependent on external environmental inputs. For prokaryotes, while the dam’s rumen is likely the dominant source of microbial transmission, other maternal niches such as milk, oral cavity, and teat skin can also contribute to early rumen colonization (4). Moreover, calves raised with their mothers acquired a broader range of bacterial genera, particularly fibrolytic taxa such as *Ruminococcus*, and viruses targeting these hosts were not only more frequently shared but also exhibited higher abundances. This suggests that maternal contact promotes the establishment of ruminal characteristics such as fermentation and subsequent VFA production, followed by increased average daily gain (Fig. S10), consistent with previous findings (5). However, dry matter intake in calves was not measured, and therefore, we cannot exclude the possibility that differences in dry matter intake between the two groups contributed to the observed differences.

Nevertheless, even in calves raised with their mothers, the proportion of shared vOTUs between cow-calf pairs did not exceed 50%. One possible explanation is that rumen viruses undergo rapid mutation, so that even when transmission occurs, sequence divergence quickly obscures detectable sharing. This hypothesis is consistent with previous studies reporting that rumen DNA viromes tend to be individual-independent (9–11). By contrast, prokaryotes showed a different pattern: at TP2 and TP3, more than 50% of MAGs were shared between dams and calves, and this level of sharing was observed even when calves were separated from their mothers. This suggests that, while some prokaryotic lineages are inherited from the dam, the establishment of the calf rumen microbiome is strongly shaped by external environmental inputs that are common across individuals, regardless of maternal contact. Together, these results highlight the contrasting transmission dynamics of prokaryotic and viral communities and emphasize that external environmental factors play a critical role in shaping the early-life rumen microbiome.

Finally, although not the primary focus of this study, our comparison between whole and VLP metagenomes revealed substantial differences in the recovery of viral diversity. VLP sequencing consistently captured a far greater number of vOTUs than whole metagenomes and largely encompassed the viral diversity detected by the latter. These findings are consistent with previous observations in soil viromes (26) and corroborate an earlier report from the rumen (9), providing additional support for differences between VLP and whole metagenomes. Notably, most studies linking rumen viromes to production traits such as milk yield or feed efficiency have relied exclusively on whole metagenomes (13, 33), whereas VLP sequencing has only recently been applied to investigate viral associations with carcass traits in beef cattle (11). Taken together, our results highlight the limitations of whole-metagenome approaches and underscore the potential of VLP sequencing to provide a more comprehensive view of rumen viral diversity, thereby offering new opportunities for linking the virome to host phenotypes in future studies.

Several limitations should be considered when interpreting the present results. First, calves in the S group received colostrum sucked from Holstein cows and were fed milk replacer, whereas calves in the C group received maternal colostrum and natural suckling. Therefore, differences in colostrum and milk sources may have independently influenced early-life rumen microbial colonization. For example, although a larger number of MAGs and VCs were enriched in the C group at TP2 and TP3, the opposite trend was observed at TP1, where more MAGs and VCs were enriched in the S group. This may have reflected microbial exposure associated with Holstein colostrum and milk replacer during the early postnatal stage. However, because microbiota from non-rumen sources, such as colostrum, milk replacer, maternal oral microbiota, and the surrounding environment, were not analyzed in the present study, the origin of these microorganisms could not be determined conclusively. Second, the maternal rumen microbiome was analyzed only at TP1. Although previous studies based on 16S rRNA gene sequencing have suggested that the rumen microbiome remains relatively stable throughout lactation (34), temporal changes in individual MAGs and viral genomes may still occur during the postpartum period. Therefore, comparisons between the maternal and calf microbiomes at TP2 and TP3 may not fully reflect maternal-calf microbial relationships over time. Third, the present study included only 11 cow-calf pairs, which may have limited the recovery of microbial diversity and reduced statistical power. As a result, our analyses were restricted to 694 MAGs and therefore did not capture the complete rumen microbial community. Consequently, some taxa, particularly low-abundance microorganisms, may have been overlooked. Together, future studies using more controlled experimental designs and larger cohorts will be important for disentangling the individual contributions of maternal contact, diet, and environmental exposure to rumen microbiome establishment.

We substantially expanded the genomic landscape of rumen prokaryotic and viral diversity in early-life calves and their dams. By reconstructing 694 MAGs and 30,479 vOTUs, we provide a genomic resource for studying rumen microbiome ecology, which will serve as a valuable reference for the global research community. Additionally, we show that prokaryotic and viral communities undergo parallel age-dependent succession, indicating that viral dynamics are closely linked to the development of their microbial hosts. Notably, our results showed that calves raised with or without maternal contact exhibited distinct rumen microbial communities. This finding underscores the critical point that the early-life rumen microbiome is highly plastic and can be modulated through environmental factors, as has been proposed in previous studies (3, 35), providing opportunities for targeted interventions. Collectively, our results provide fundamental insights into the establishment and succession of the calf rumen microbiome and DNA virome during early life and may contribute to future studies aimed at microbiome manipulation in ruminants.

## MATERIALS AND METHODS

### Animals, experimental design, and sampling

Eleven Japanese Black cow-calf pairs were used. The animals were assigned to two groups: the control group (C; five cow-calf pairs), in which calves were raised together with their mothers from birth throughout the experiment, and the separation group (S; six cow-calf pairs), in which calves were separated from their mothers immediately after birth and raised without any maternal contact. Animals in groups C and S were housed in separate wheat straw-bedded pens within the same cattle barn. The information on animals is summarized in Table S1. For colostrum, calves in group C received it directly from their dams, whereas calves in group S were fed 3 kg of colostrum obtained by Holstein cows on day 1. Calves in group C continued to suckle their dams, while calves in group S were fed with commercial milk replacer (Crude protein >28.0%; Ether extract >18.0%; Crude ash >10.0%; Ca > 0.8%; *P* > 0.5%; Total digestible nutrients > 105%) after day 2. The amount of milk replacer provided was 600 g/day from days 2 to 16, 800 g/day from days 17 to 43, 600 g/day from days 44 to 50, and 400 g/day from days 51 to 70. In both groups, commercial calf starter pellet (Crude protein >20.0%; Ether extract >9.0%; Crude ash >8.0%; Ca > 0.6%; *P* > 0.4%; Total digestible nutrients >75%), Italian ryegrass, and water were provided *ad libitum* during the experiment. All animal procedures were approved by the Utsunomiya University Animal Ethics Committee (permit number: A23c-0012).

To investigate rumen microbiome development during the preweaning period, rumen liquid samples from calves were collected three times: on days 6–8 (TP1), 38–40 (TP2), and 68–70 (TP3) after birth. Rumen liquid samples from the mothers were collected on the same day as the first sampling of the calves (M). One calf in group S was removed from the experiment after the TP1 sampling due to health problems. Rumen samples were collected using an orogastric tube. The first collected sample (approximately 100 mL) was discarded to minimize saliva contamination. The rumen samples were filtered through four layers of cheesecloth and immediately placed on dry ice. The samples were stored at –80°C until analysis. Body weight (BW) of calves was measured at all time points, while BW of cows was measured once, within 1 month after delivery.

### Whole-metagenomic sequencing

One milliliter of rumen liquid was centrifuged at 12,000 × *g* for 15 min at 4°C, and the pellet was used for DNA extraction. Whole genomic DNA was extracted using a previously reported protocol involving enzymatic lysis with lysozyme and proteinase K, followed by phenol-chloroform extraction and ethanol precipitation (36). Whole-metagenome libraries were prepared using the Illumina DNA Prep Kit (Illumina, San Diego, CA, USA) and sequenced on the Illumina NovaSeq X Plus platform (2 × 150 bp).

### VLP-metagenomic sequencing

VLP purification and DNA extraction from rumen fluid were carried out following a validated protocol (11). Briefly, rumen samples were centrifuged to remove debris and sequentially filtered through 0.45-μm and 0.22-μm filters. VLPs were concentrated using polyethylene glycol precipitation, followed by DNase and RNase treatment. VLP DNA was then extracted using proteinase K treatment and phenol-chloroform extraction. The resulting VLP DNA was used for library construction and sequencing as described above.

### Analysis of the prokaryotic community

Low-quality reads and adapter sequences from whole-metagenome sequencing were removed using Trimmomatic v0.39 (37). Host-derived sequences were filtered out by aligning trimmed reads to the bovine reference genome (GCF_002263795) with BWA-MEM (38), and only unmapped reads were retained. After quality filtering and host read removal, 78.5% ± 8.2% of reads were retained for downstream analyses. Filtered paired and unpaired reads from each sample were independently assembled using SPAdes v3.15.3 with the “--meta” option (39). Contigs longer than 1,000 bp were retained for subsequent analyses.

Filtered reads were mapped back to the contigs with BWA-MEM (38) for binning. Binning was performed using MaxBin2 v2.15 (40), MetaBAT2 v2.27 (41), and CONCOCT v1.10 (42), and the resulting bins were refined using DAS Tool v1.1.4 (43). MAG quality was assessed with CheckM2 v1.0.2 (16), and only “medium-quality” (≥50% completeness and ≤10% contamination) and “high-quality” MAGs (≥90% completeness and ≤5% contamination) were retained. These MAGs were dereplicated at 99% ANI using dRep v3.2.2 (17), and non-redundant MAGs were used for downstream analyses.

Taxonomic classification of MAGs was conducted using GTDB-Tk v2.4.0 (18) with GTDB release 226. A phylogenetic tree was constructed with PhyloPhlAn v3.0.60 (44) and visualized in iTOL v7.1 (45). Gene prediction was carried out with Prodigal v2.6.3 (46), and genes with partial ORFs were excluded. CAZyme annotation was performed using HMMER v3.3 (47) against the dbCAN HMM v10 database, applying thresholds of E-value <1e−15 and coverage >0.35 (48).

Filtered paired reads were mapped to the non-redundant MAGs using CoverM v0.4.0 (https://github.com/wwood/CoverM) with the parameters --trim-min 0.10 --trim-max 0.90 --min-covered-fraction 0 --min-read-percent-identity 95 --min-read-aligned-percent 75. MAGs with <10% genome coverage were considered absent (count = 0). Read counts were normalized to transcripts per million (TPM) values for subsequent analyses.

### Data processing and vOTU clustering

VLP-metagenomic data were filtered and assembled as described above. On average, 88.5% ± 5.6% of reads passed quality filtering and host read removal. Contigs longer than 5 kb from both whole and VLP metagenomes were collected. Viral contigs were identified with geNomad v1.8.0 (19), and their completeness was assessed using CheckV v0.8.1 (20). Viral candidates classified as “complete,” “high-quality” (≥90% completeness), or “medium-quality” (50%–90% completeness) were pooled across samples and clustered at 95% nucleotide identity and 85% alignment coverage using CD-HIT v4.8.1 (49) to define vOTUs.

Protein-coding genes in vOTUs were predicted with Prodigal v2.6.3 (46), and partial ORFs were removed. vOTUs were further grouped into VCs using vConTACT2 v0.9.19 (21) with the ProkaryoticViralRefSeq201-Merged database. vOTUs classified as “outliers” or “overlaps” were regarded as singletons, which were each treated as an independent VC. To assess the novelty of the reconstructed viral genomes, RCCV vOTUs were compared with previously published rumen viral genome collections, including RVD (*n* = 41,738) (9), RVG (*n* = 8,232) (10), and JBRV (*n* = 22,942) (11). RCCV and reference viral genomes were clustered together using CD-HIT v4.8.1 (49) at 95% nucleotide identity and 85% alignment coverage. Taxonomy was assigned with geNomad (19), and viral lifestyle was predicted with PhaTYP (50).

Virus-prokaryote interactions were inferred from CRISPR spacers. Spacers with evidence levels 3 or 4 were extracted from reconstructed MAGs using CRISPRCasFinder v4.2.20 (51). These spacers, along with the RuSpacer database (22), were aligned against vOTUs using blastn-short from BLAST + v2.9.0 (52) with the following thresholds: ≥95% nucleotide identity, ≥95% query coverage, and *E*-value ≤1e−3. Matches with ≤1 combined mismatch and gap were considered high confidence.

Filtered paired reads were mapped to vOTUs using CoverM with the same criteria for MAGs. vOTUs with <70% covered fraction in a sample were considered absent (count = 0). TPM values were then calculated per vOTU and summed within each VC to estimate VC abundances.

### Statistical analyses

For beta-diversity analyses, count matrices were transformed using the centered log-ratio transformation with a pseudocount of 0.01, and Euclidean distances were calculated. Community differences among TP1, TP2, TP3, and M were assessed using PERMANOVA followed by pairwise PERMANOVA comparisons. *P* values from pairwise comparisons were adjusted using the Benjamini–Hochberg FDR correction. Differences between groups C and S as well as between VLP- and whole-metagenomic viral communities at each time point were evaluated using PERMANOVA. All analyses were performed using the vegan package (53). For alpha-diversity, the numbers of observed MAGs and vOTUs were compared among TP1, TP2, TP3, and M, between groups C and S, and between VLP- and whole-metagenomic data sets using the Wilcoxon rank-sum test. *P* values were adjusted for multiple comparisons using the Benjamini–Hochberg FDR correction.

Differences in the abundance of MAGs and VCs between groups C and S were tested using DESeq2 (28), with raw count matrices as input. Features detected in at least 50% of samples in either group were retained for analysis, and those with an absolute log2 fold change > 2 and FDR < 0.05 were considered significantly different. Age-dependent changes in MAG and VC abundances were evaluated using MaAsLin2 (27), with centered log-ratio-transformed values. Only calf samples from TP1 to TP3 were included, and features detected in at least 10% of samples were retained. Time was treated as a continuous fixed effect and Animal as a random effect. Features with FDR < 0.05 were considered significant.

## Data Availability

Raw sequence data were deposited in DDBJ (accession number: PRJDB37641. Non-redundant MAGs and vOTUs are available in figshare (https://figshare.com/s/d9e511b00f6c86a27fbc). The code used for the analysis is available on GitHub (https://github.com/aki0514/Rumen-metagenome-scripts).

## SUPPLEMENTAL MATERIAL

Supplemental figures spectrum.01677-26-s0001.pdf Fig. S1 to S12.

Table S1 spectrum.01677-26-s0002.xlsx Summary of information about the animals.

Table S2 spectrum.01677-26-s0003.xlsx Overview of the MAGs reconstructed in this study.

Table S3 spectrum.01677-26-s0004.xlsx Summary of vOTUs.

ASM does not own the copyrights to Supplemental Material that may be linked to, or accessed through, an article. The authors have granted ASM a non-exclusive, world-wide license to publish the Supplemental Material files. Please contact the corresponding author directly for reuse.
