# Supplementary material for: Maternal contact and age-dependent succession influence the assembly of the calf rumen microbiome and virome
Source: Microbiol Spectr. 2026 Jul 22;14(9):e01677-26. doi: 10.1128/spectrum.01677-26 (PMC13532254; doi:10.1128/spectrum.01677-26)
Supplement: Supplementary file 1 [file spectrum.01677-26-s0001.pdf]

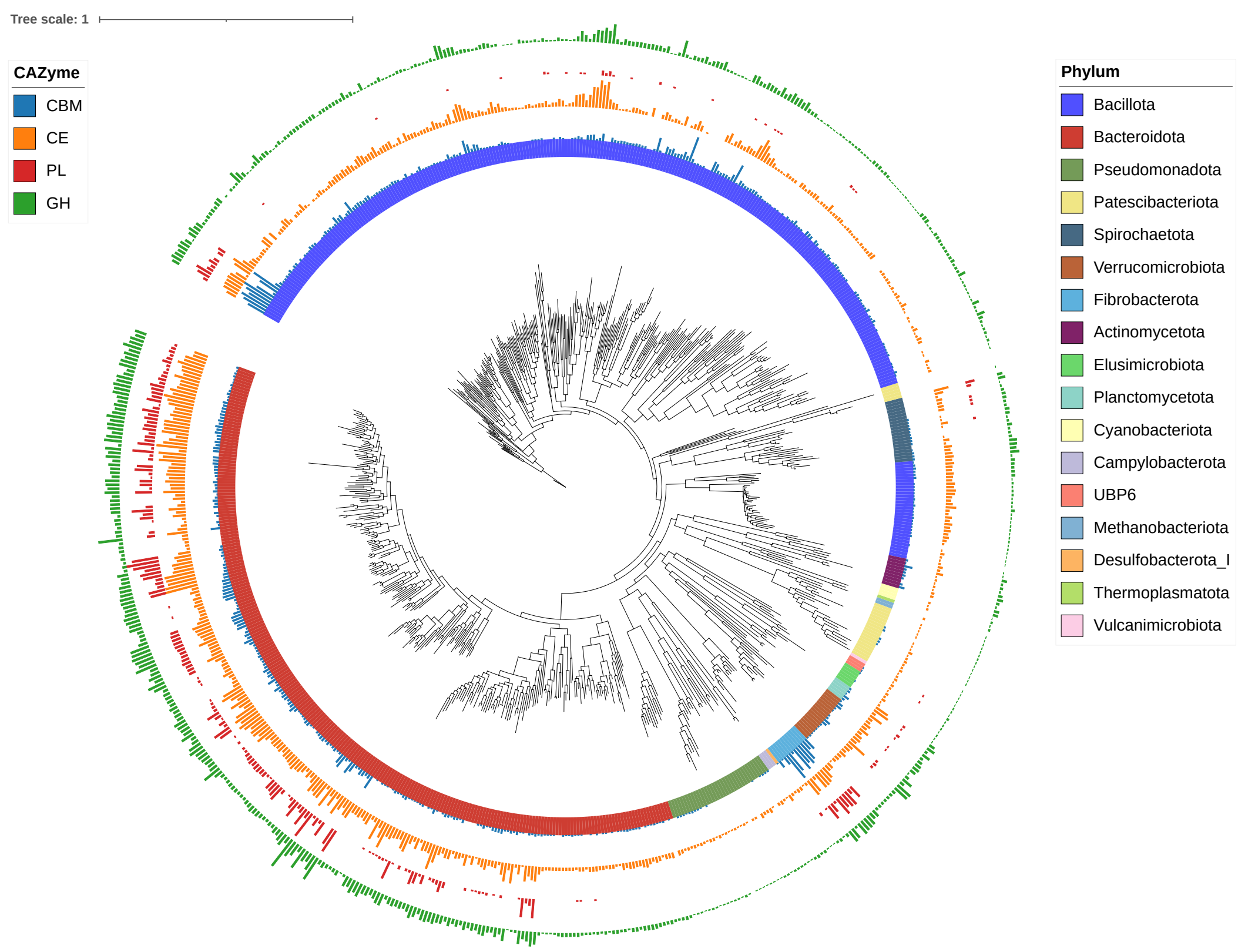

Fig. S1 Phylogenetic tree of 694 reconstructed MAGs from the rumens of calves and cows.

The first outer ring represents phylum-level taxonomy, with different colors assigned to each phylum. Beyond this, four concentric rings indicate the number of CAZymes in each category: GH (Glycoside Hydrolases), CE (Carbohydrate Esterases), PL (Polysaccharide Lyases), and CBM (Carbohydrate-Binding Modules).

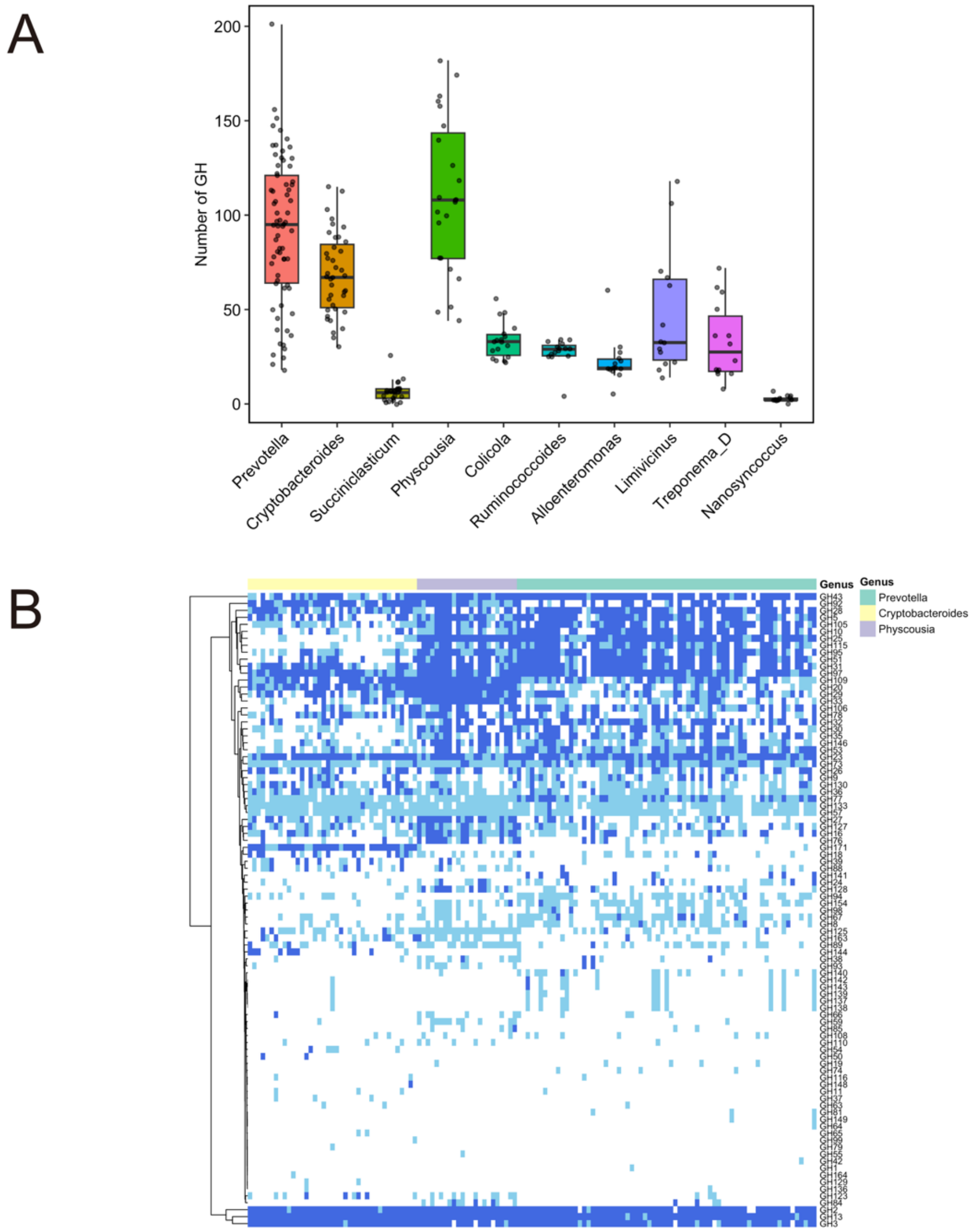

Fig. S2 Distribution of Glycoside Hydrolases (GHs).

(A) The number of GHs in the top 10 genera. (B) Heatmap showing the distribution of individual GH families across the main rumen genera. Dark blue indicates ≥2 occurrences, and light blue indicates 1 occurrence.

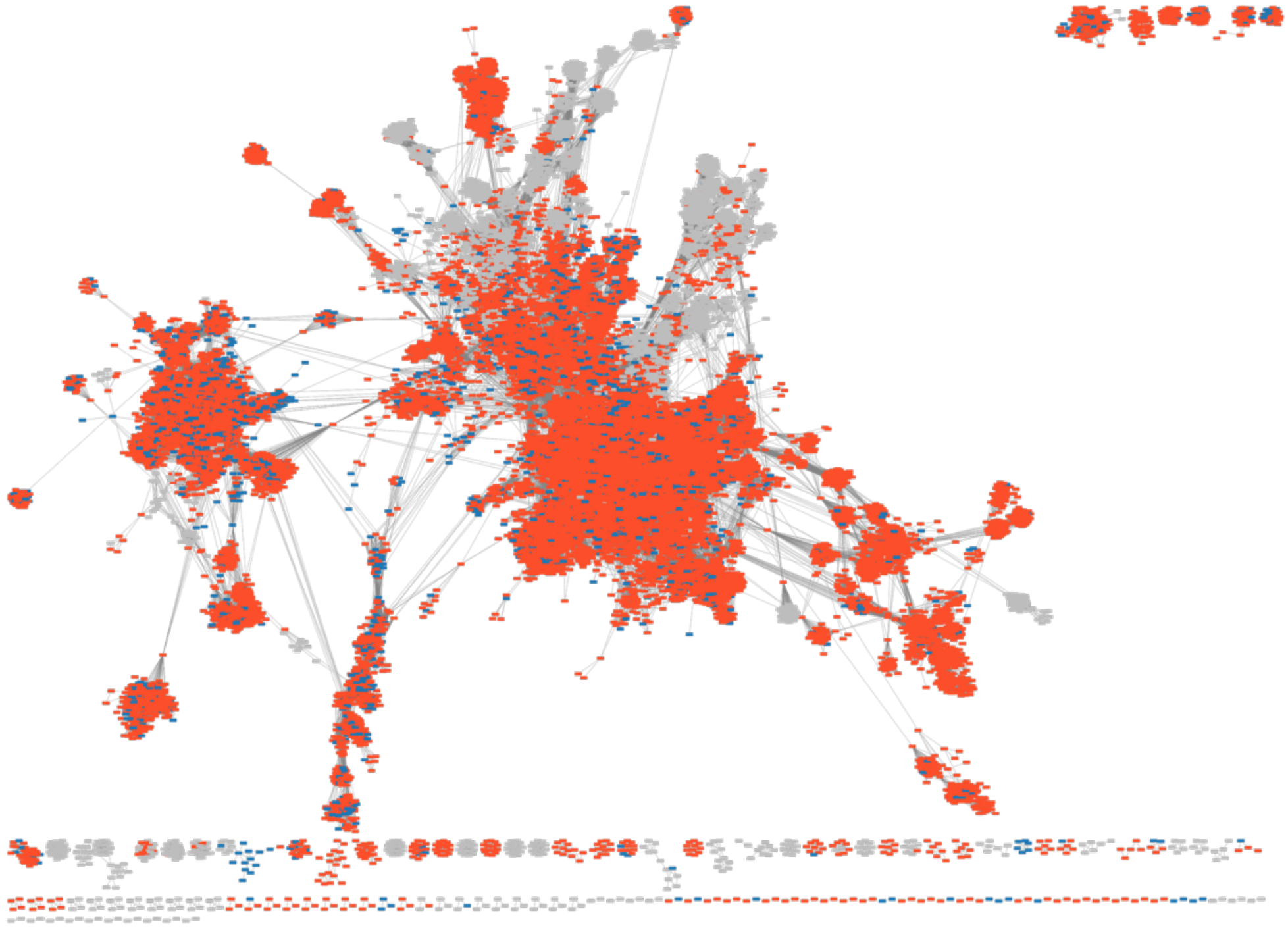

Fig. S3 Gene-sharing network of viral genomes.

Each dot represents a viral genome, and lines indicate gene-sharing connections between genomes. Red and blue nodes correspond to viral genomes from the RCCV dataset obtained by VLP- and whole- metagenomic sequencing, respectively, while grey nodes represent viral genomes from RefSeq.

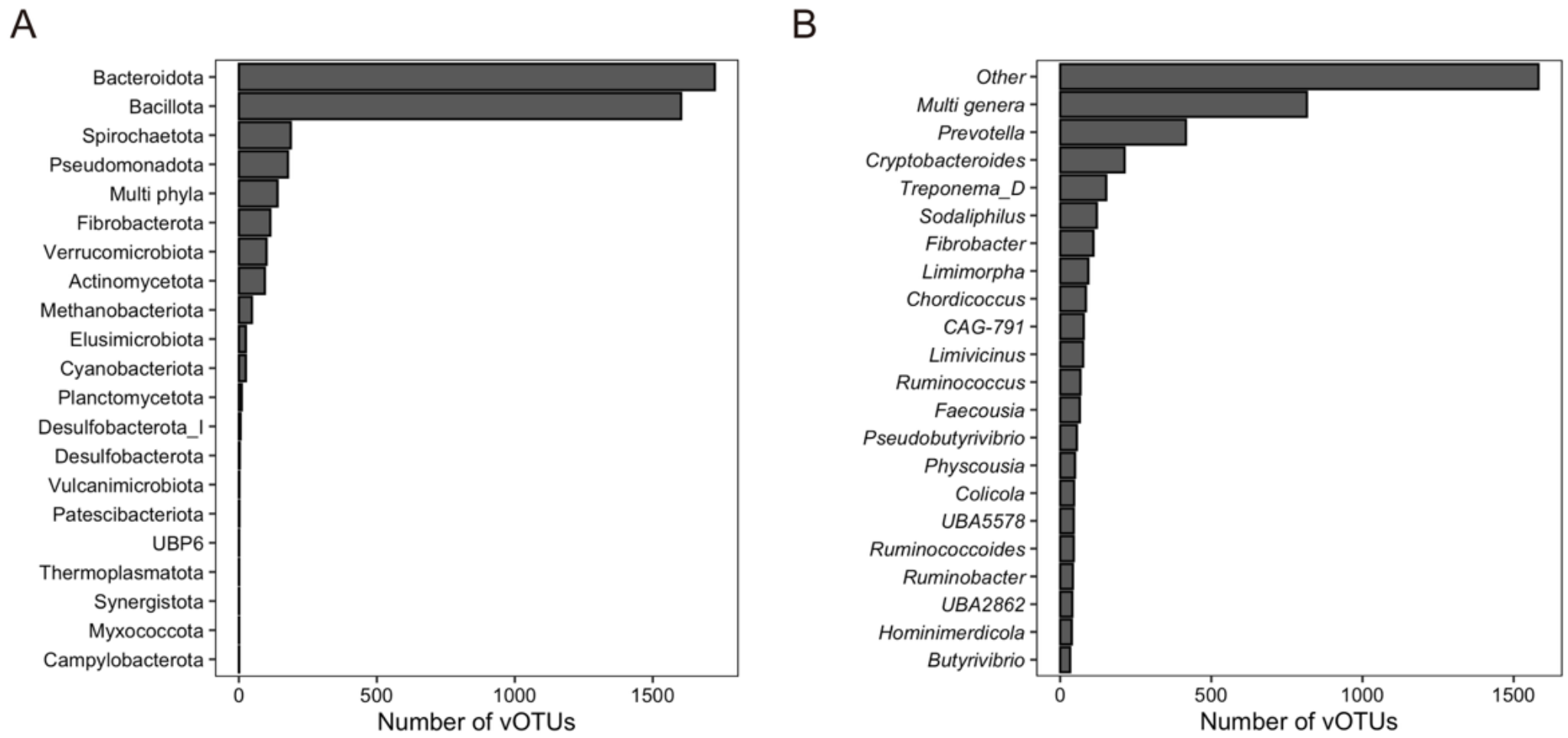

Fig. S4 Host–virus interactions in RCCV.

The number of predicted hosts at (A) the phylum level and (B) the genus level.

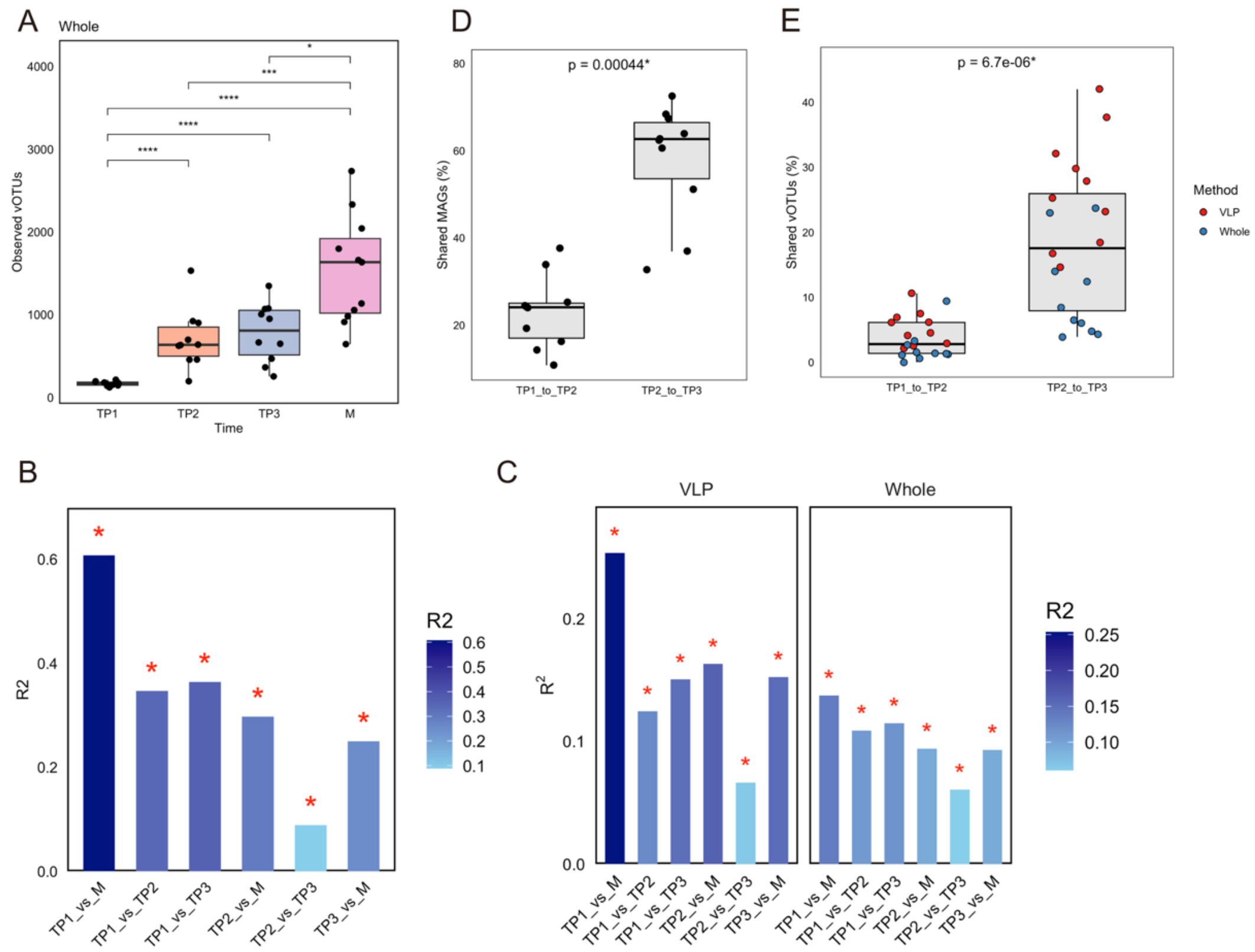

Fig. S5 Time-dependent developments of prokaryotic and viral communities. (A) Number of observed vOTUs using whole-metagenome sequencing. Statistical significance was assessed by Wilcoxon rank-sum tests with false discovery rate (FDR) correction and indicated as follows: FDR ≤ 0.05 (*), FDR ≤ 0.001 (***), FDR ≤ 0.0001 (****). (B, C) PERMANOVA analysis of MAGs and vOTUs across all time points (TP1–TP3) and mother (M). Red asterisks indicate significant differences (FDR < 0.05). (D, E) Shared percentages of MAGs and vOTUs between TP1 and TP2, and between TP2 and TP3.

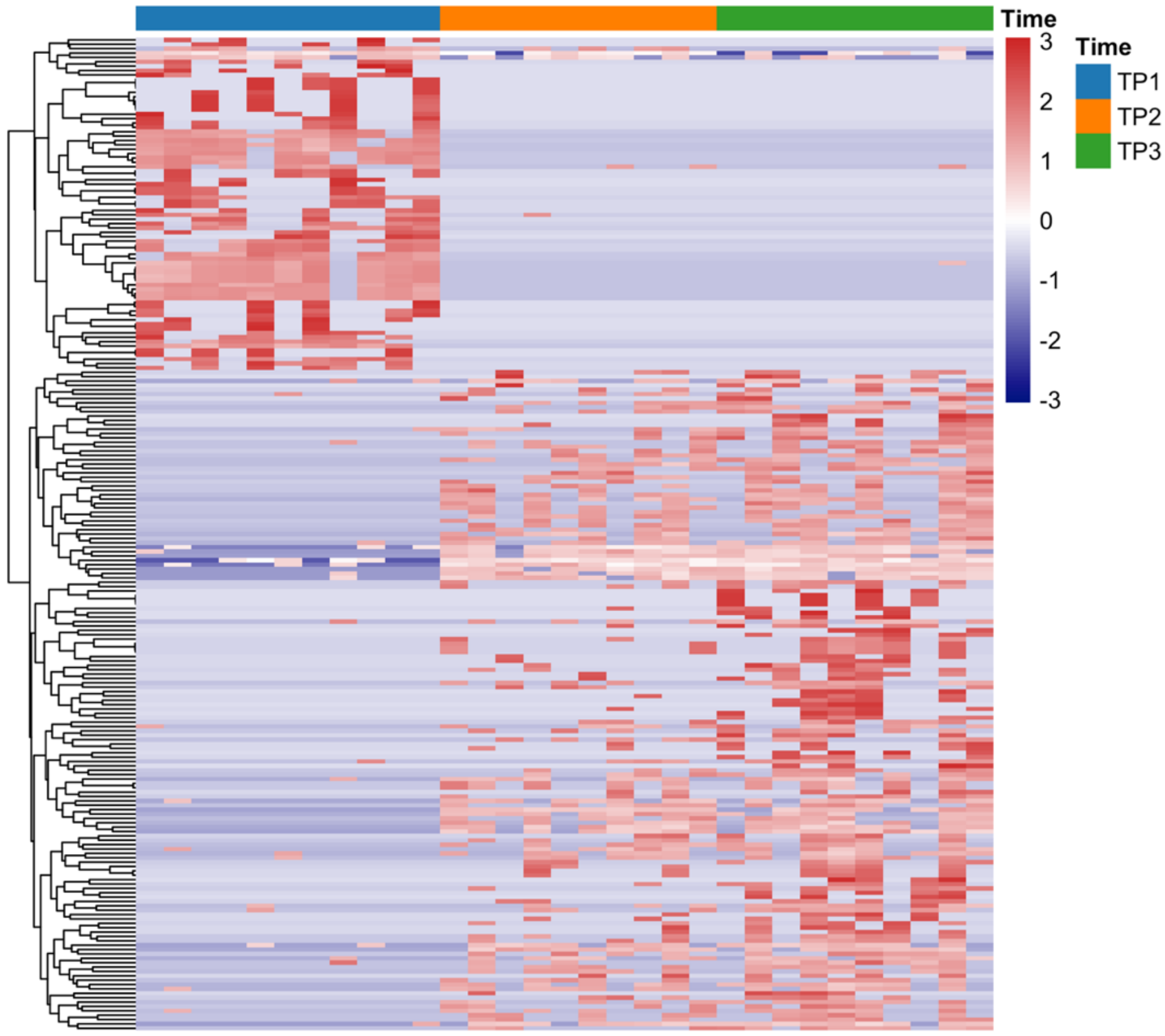

Fig. S6 Heatmap of significant time-dependent vOTUs identified using whole-metagenome sequencing. Statistical analysis was conducted using MaAsLin2, and vOTUs with FDR < 0.05 were considered significant. Values represent CLR-transformed TPM (TPM + 0.01).

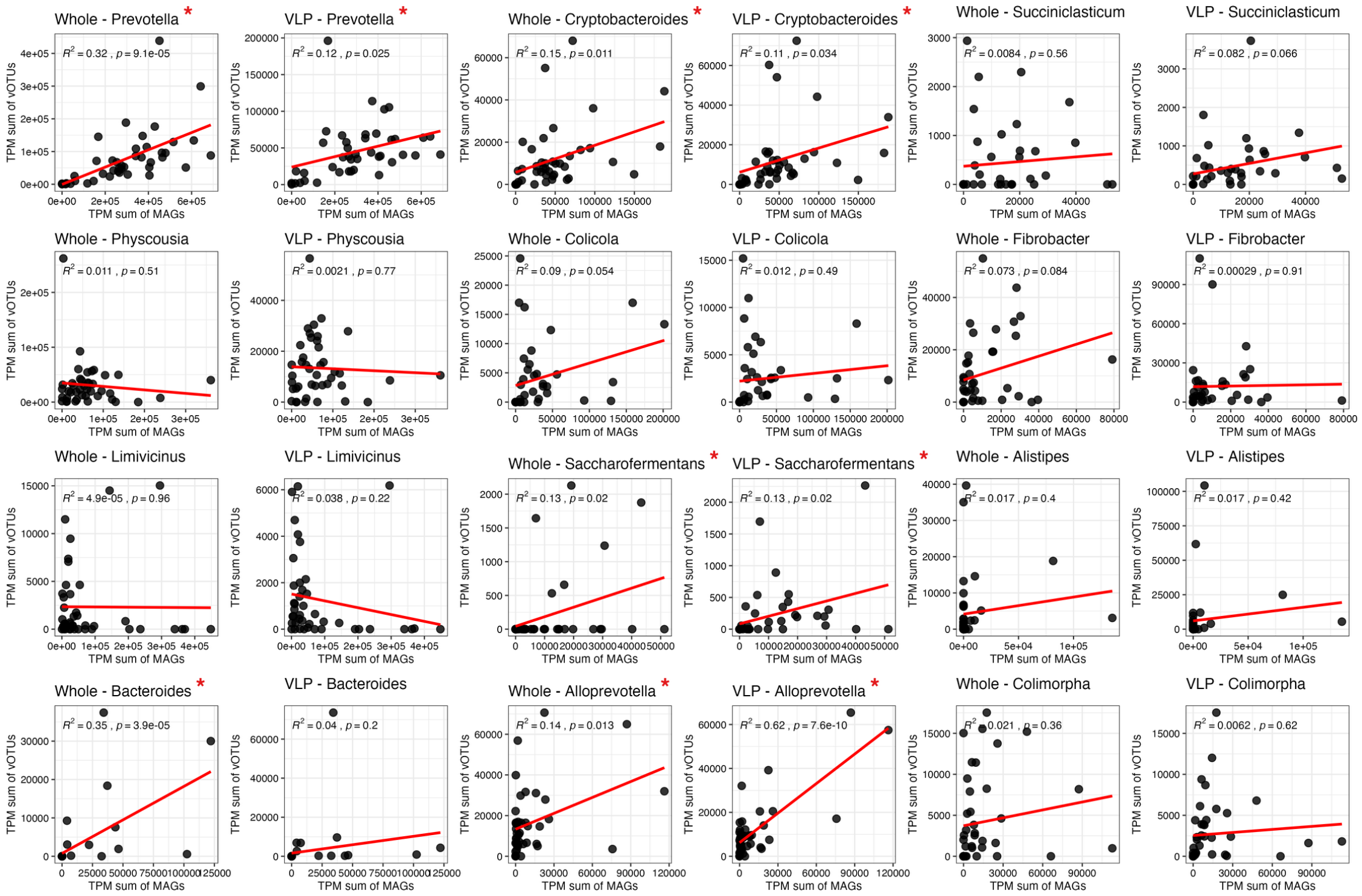

Fig. S7 Pearson correlation analysis between vOTUs and their predicted hosts at the genus level. Red asterisks indicate significant correlations ($p < 0.05$).

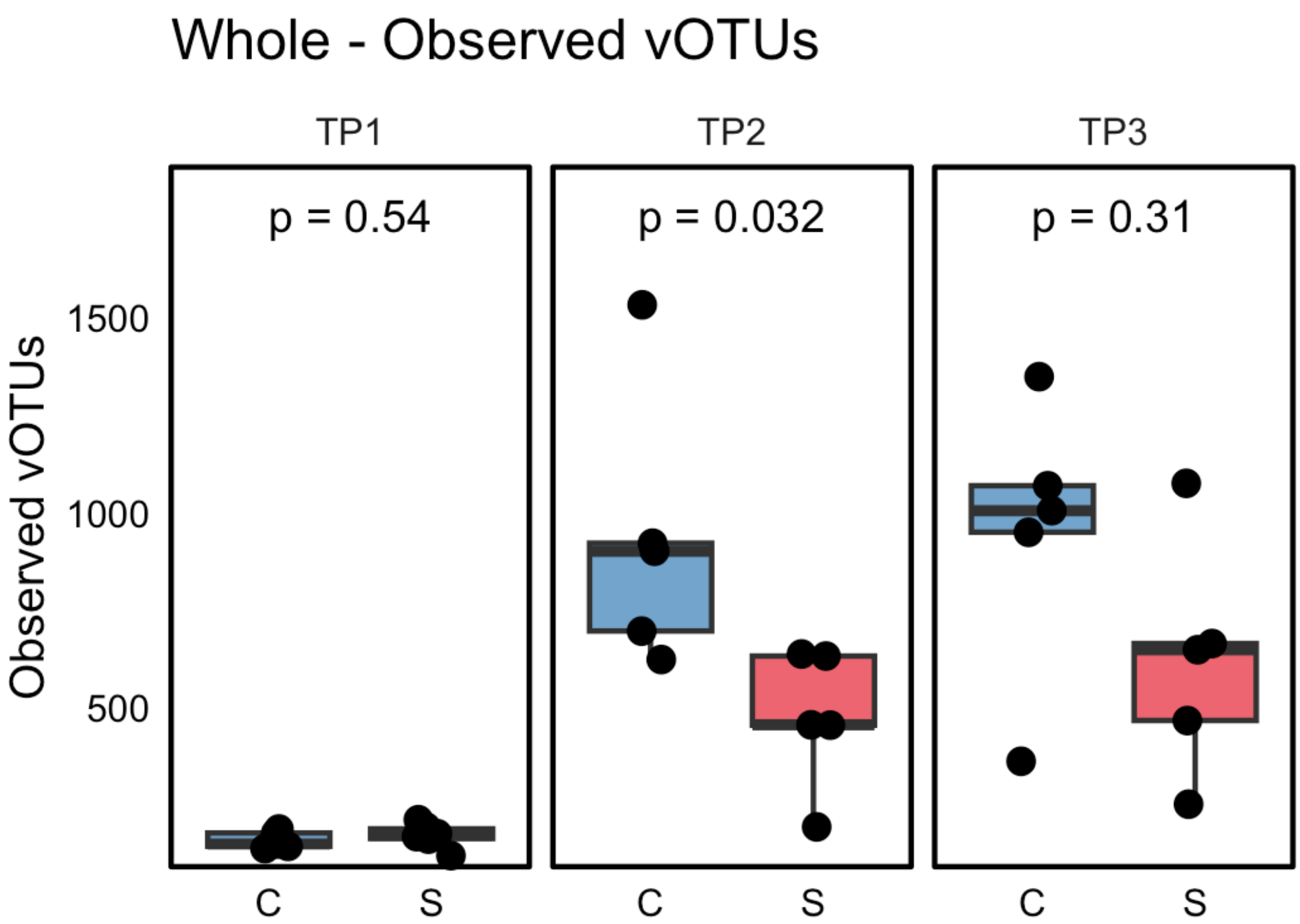

Fig. S8 Differences in the number of observed vOTUs between groups C and S using whole-metagenome sequencing.

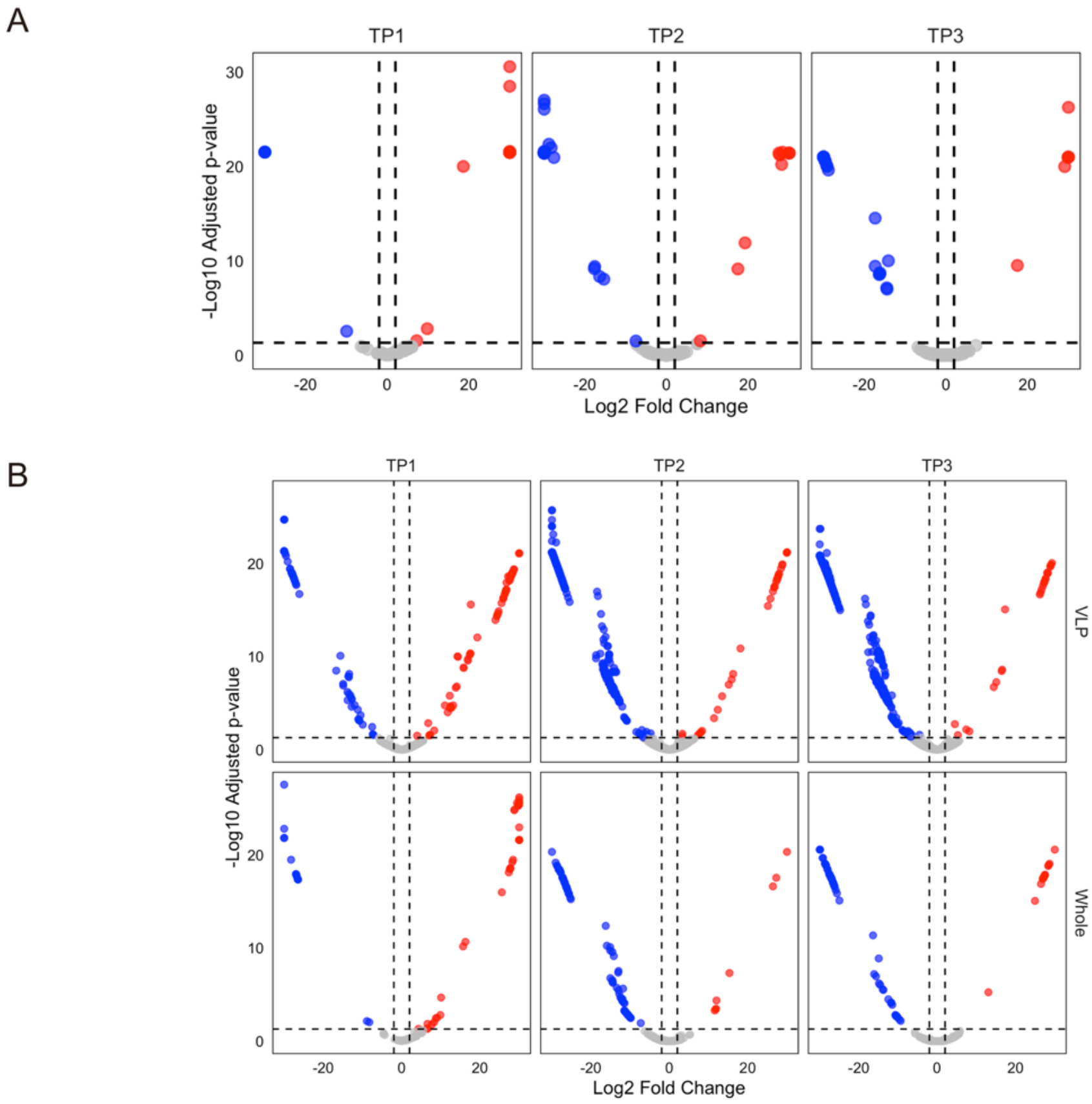

Fig. S9 Volcano plots showing differences in the microbiome. Blue and red dots represent statistically significant (A) MAGs and (B) vOTUs enriched in groups C and S, respectively (FDR < 0.05 and |log2 fold change| > 2). The analysis was performed using DESeq2.

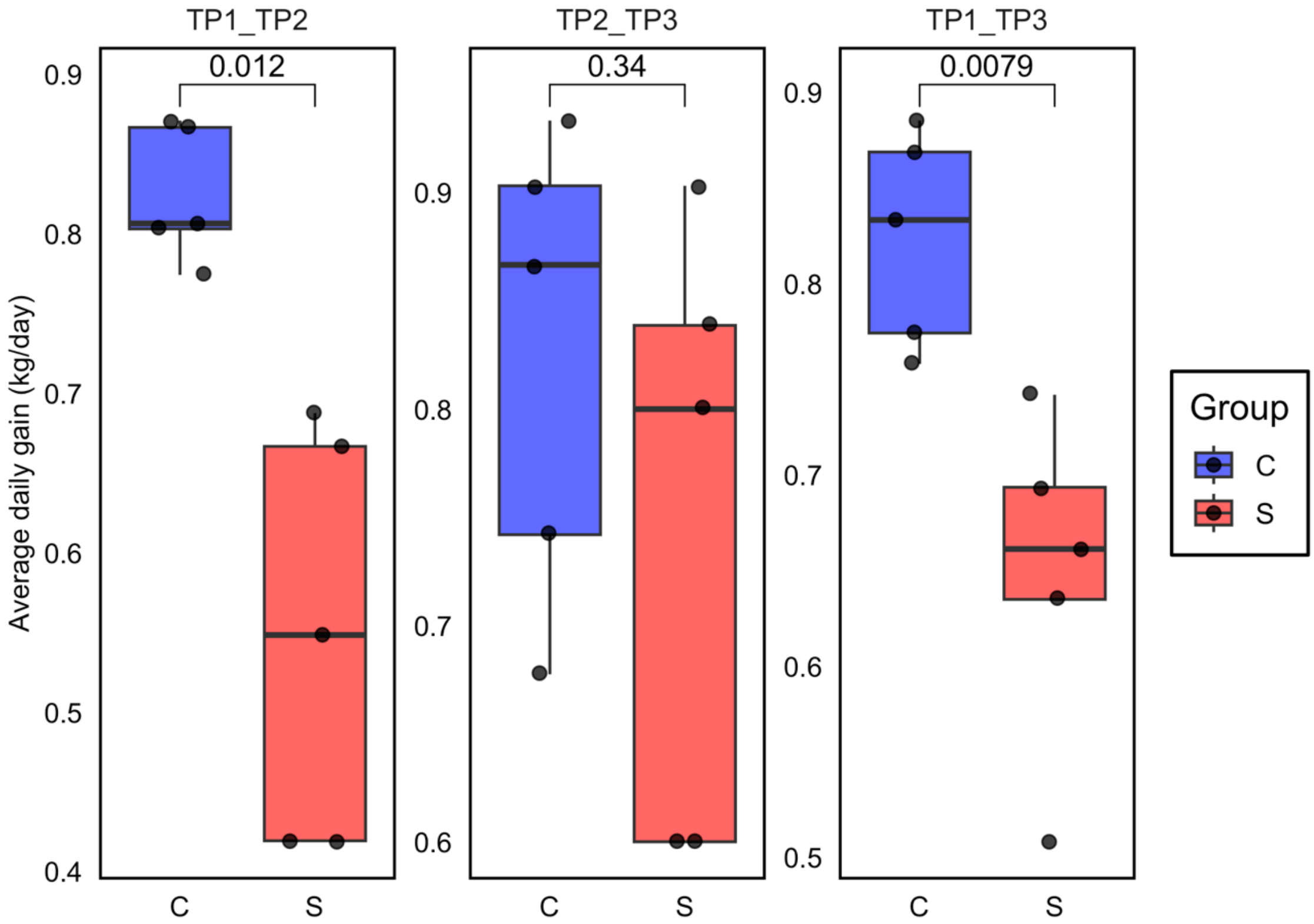

Fig. S10 Differences in average daily gain of animals between groups C and S.

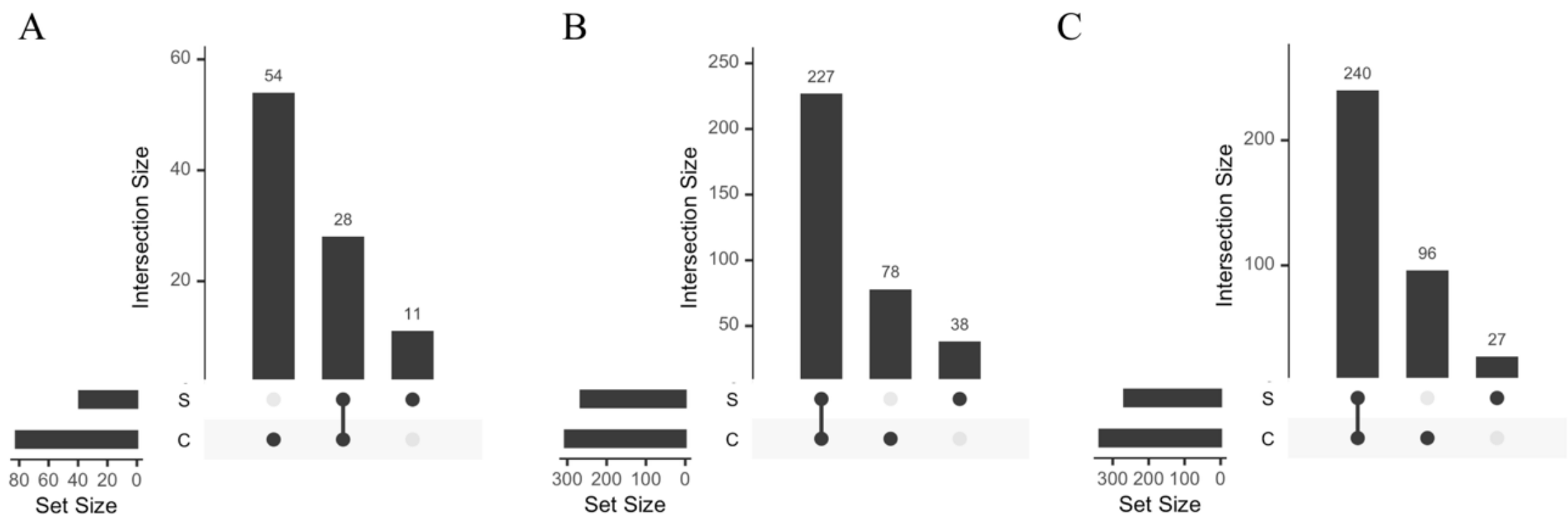

Fig. S11 Differences in shared MAGs between groups C and S. UpSet plots showing shared MAGs between cow–calf pairs at (A) TP1, (B) TP2, and (C) TP3.

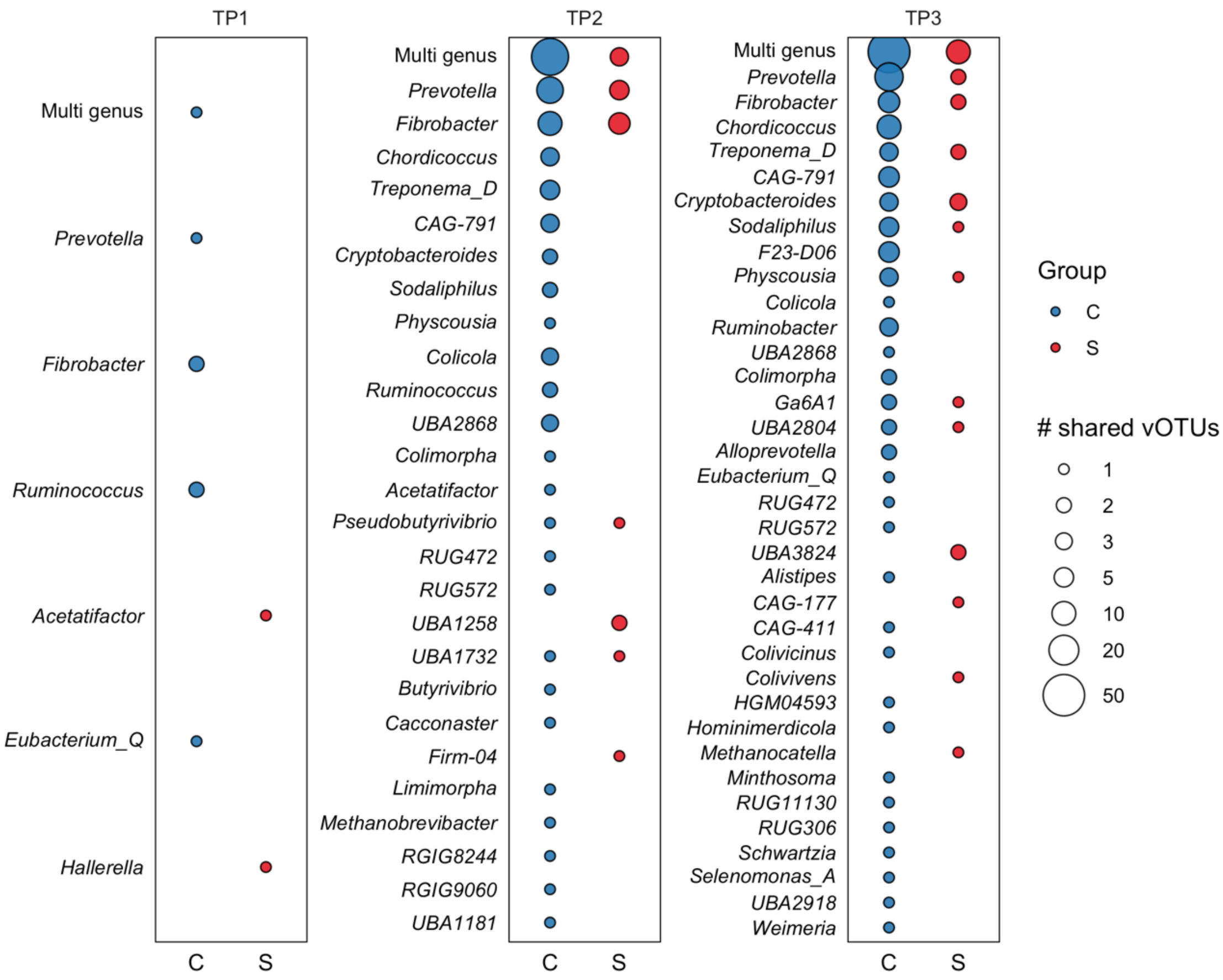

Fig. S12 Predicted hosts of shared vOTUs between cow–calf pairs in separation and non-separation groups based on whole-metagenomes.
